# A nanotrap infused ultrathin hybrid composite material for rapid and highly selective entrapment of ^99^TcO_4_^−^†

**DOI:** 10.1039/d4sc04010d

**Published:** 2024-10-08

**Authors:** Writakshi Mandal, Sahel Fajal, Dipanjan Majumder, Arijit Sengupta, Sumanta Let, Rajashri R. Urkude, Mandar M. Shirolkar, Arun Torris, Sujit K. Ghosh

**Affiliations:** a Department of Chemistry, Indian Institute of Science Education and Research (IISER) Pune Dr Homi Bhaba Road, Pashan Pune 411 008 India sghosh@iiserpune.ac.in; b Radiochemistry Division, Bhabha Atomic Research Centre Mumbai 400085 India; c Homi Bhabha National Institute Mumbai 400094 India; d Beamline Development and Application Section Bhabha Atomic Research Centre Mumbai 400085 India; e Advanced Bio-Agro Tech Pvt. Ltd Baner Pune 411045 India; f Norel Nutrient Bio-Agro Tech Pvt. Ltd Baner 411045 India; g Polymer Science and Engineering Division, CSIR-National Chemical Laboratory Dr Homi Bhabha Road Pune 411008 India; h Centre for Water Research (CWR), Indian Institute of Science Education and Research (IISER) Pune Dr Homi Bhabha Road, Pashan Pune 411 008 India

## Abstract

^99^Tc is one of the potentially toxic radioactive substances owing to its long half-life and a high degree of environmental mobility. Hence, the sequestration of ^99^Tc from radioactive waste has become enormously important and a contemporary research priority. However, selective extraction of this species in its stable oxoanionic form (^99^TcO_4_^−^) is very challenging on account of bottlenecks such as low charge density, less hydrophilic nature, *etc.* Herein, an ultrathin hybrid composite material has been strategically designed and fabricated by covalent anchoring of a chemically stable amino functionalized nanosized cationic metal–organic polyhedron with a positively charged robust ionic covalent organic framework. The resulting thin-layer-based hybrid composite presented multiple exfoliated exposed interactive sites, including a Zr(iv)-secondary building unit, amine and triaminoguanidine functional groups, which can selectively interact with TcO_4_^−^ oxoanions through a synergistic combination of electrostatic, H-bonding and various other supramolecular interactions. Thus synthesized function-tailored composite, by virtue of its multiple unique characteristics, manifested an ultrafast and very selective, high distribution coefficient (∼10^6^ mL g^−1^), as well as recyclable entrapment of TcO_4_^−^ oxoanions from the complex mixture of superfluous (∼5000-fold) other interfering anions in both high and ultra-trace concentrations along with simulated nuclear waste and from different water systems. Dynamic flow-through experiments were conducted with the membrane of the hybrid material in simulated wastewater, which reduced the concentration of ReO_4_^−^ (surrogate of radioactive TcO_4_^−^) to below the WHO permissible level with rapid sequestration kinetics and excellent selectivity over excessive competing anions.

## Abstract 2

A multifunctional ultrathin hybrid nanoadsorbent demonstrated ultrafast and highly selective sequestration of toxic radioanions such as ^99^TcO_4_^−^ by both batch and dynamic column exchange methods from simulated nuclear waste and contaminated water.

## Graphical abstract

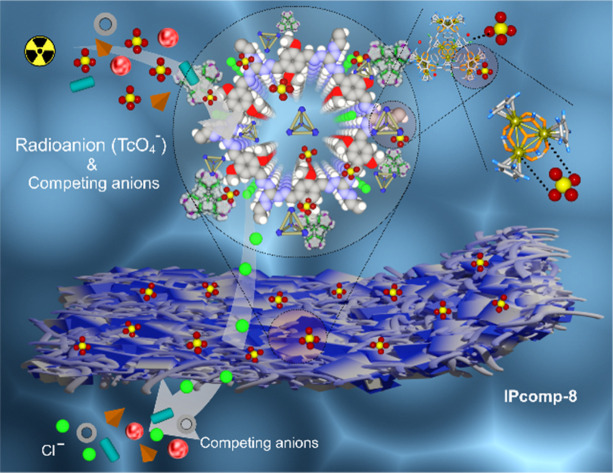

## Introduction

**Scheme 1:**
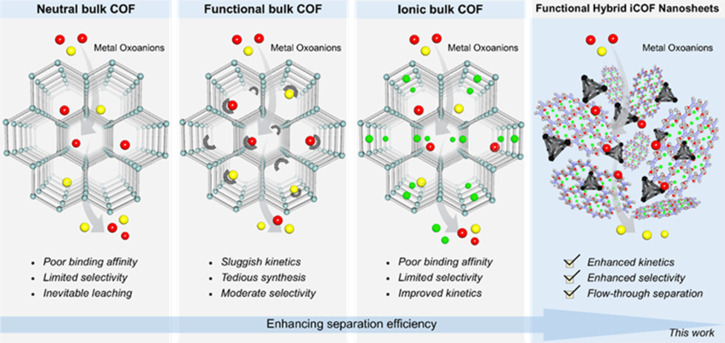
Schematic illustration of the advantages of MOPs@iCOF hybrid composite nanosheet materials over pristine neutral bulk COF and functionalized and ionic COF materials, towards rapid and selective oxoanion sequestration.

^99^Tc prevails as one of the most hazardous fission products in the nuclear fuel cycle because of its long half-life, potential radiation threat, high environmental mobility and strong redox-active nature.^1^^99^Tc is a long lived (*t*_1/2_ = 2.13 × 10^5^*a*) β-emitting radionuclide (*E* = 294 keV) that is both chemically toxic and hazardous.^2–4^ The low charge density and high symmetry of the oxoanionic species of ^99^Tc (^99^TcO_4_^−^) result in its extremely high solubility in water (11.3 M at 20 °C).^5^ Hence, ^99^TcO_4_^−^ can easily migrate through the natural water system and its migration is not affected by the abundance of the majority of natural materials. Therefore, attempts to develop several effective technologies toward sequestration and management of radioactive waste from water are governed by concerns about the environment and human health as well as the establishment of a safe nuclear sector. Among the explored technologies, solid sorbents operating *via* adsorption followed by ion exchange have demonstrated significant promise as established TcO_4_^−^ remediation technologies due to their easy, safe, low-cost and robust mechanism processes.^6–12^ Along this line, several materials, that include ion-exchange resins and polymeric networks,^13,14^ inorganic cationic materials, and cationic metal–organic frameworks (MOFs),^15^ have been explored as ^99^TcO_4_^−^ segregating materials. Albeit, most cases do not present the desired combination of a suitable sorbent material and are often associated with either suboptimal capacity^12,16,17^ and selectivity^18^ or poor removal kinetics.^12^ Therefore, the overall efficiency of these materials as potential adsorbents is limited, particularly due to their poor selectivity. This is because plenty of co-existing competing anions, such as NO_3_^−^, SO_4_^2−^, CO_3_^2−^, ClO_4_^−^, Br^−^ and Cl^−^, are in large excess with TcO_4_^−^, which can act as interfering analytes, in both natural water systems and nuclear wastewater.^13–15^ On the other hand, although commercially available polymeric anion exchange resins have a significant advantage in TcO_4_^−^ uptake selectivity, their poor radiation resistance has thwarted their practical applicability.^19^ Additionally, TcO_4_^−^ can be extracted from aqueous solution with better adsorption kinetics, uptake capacity, reasonable selectivity, and strong radiation resistance capability using cationic metal–organic frameworks (MOFs).^15,20–22^ However, the major disadvantage associated with these materials is their instability in chemical environments essential for practical use. Consequently, considerable attention has been paid to the development of effective regenerable materials with significantly better segregation capability in order to overcome these issues. Having said that, although covalent organic frameworks (COFs), in various capacities such as neutral/unmodified, ionicity or chelating functional groups have shown capability toward extracting toxic metal oxo-anions from water they are thwarted from real time utility by their moderate sorption capacity as well as suboptimal trapping kinetics and selectivity (Scheme 1).^10,23–27^ Therefore, as an ongoing battle, improvement in terms of rapid kinetics, high selectivity and capacity towards TcO_4_^−^ oxoanions at both high and ultra-trace concentrations, from nuclear wastewater sources to point-of-use, is still needed for practical application, which is mostly unexplored due to the lack of rational synthetic design strategies.

In this sense, innovative functional porous materials have been identified as potential grounds for a variety of cutting-edge applications due to their predesigned adjustable reticular structure. The synthesis of composite materials can result in the combined benefits and maximized performance of several individual functional materials.^28–31^ Toward the development of innovative and multipurpose platforms in order to achieve desired applications, hybrid composite materials have come to the forefront and garnered a lot of research attention globally.^32–34^ Although, there has been remarkable advancement in the field of composite materials in recent years,^35^ hybrid composite materials have not been that much explored yet.^36^ Along this line, the combined qualities of two individual active porous material based hybrids enable the composite to function as an excellent adsorbent.^37^ In this context, metal–organic polyhedra (MOPs), zero-dimensional crystalline materials, made up from metal ions (or metal clusters) and organic linkers, and covalent organic frameworks (COFs), crystalline porous organic materials which are assembled from covalent linking of pure organic linkers, could be an ideal platform as two individual porous materials for the hybrid.^38,39^ These two are widely known porous materials for a variety of applications such as gas storage, sensing, catalysis, photoelectric conversion, energy storage and biological activities.^38–40^ However, due to unavoidable aggregation-induced active site obstruction upon guest removal in the solid-state, MOPs are often unable to attain their full potential in the aforementioned applications.^41^ To date, only a few strategies have been documented to overcome such a prime issue by encasing MOPs inside porous materials (silica and MOFs)^42,43^ but hybridization of MOPs with other porous materials such as COFs has remained a difficult challenge. Nevertheless, covalent organic framework (COF) powder hybridized with cationic cages has been found to exhibit improved kinetics and moderate selectivity in the removal of different oxoanions from water.^36^ However, the intrinsically two-dimensional stacked architecture of the neutral COF matrix of the nanocomposite limits the full exposure or utilization of their complete surface for adsorption, especially towards the active nanotraps present inside the pores. Moreover, the robust heterogeneous nature with non-monolithic applicability of bulk hybrid COF powder has thwarted the large-scale practical flow-through separation application. Now, to address these cardinal issues, we reinvestigated the molecular-level engineering of the host matrix to fabricate an effective composite material for highly efficient metal-oxoanion sequestration. In this regard, the construction of selective binding site containing active nanotrap (MOP) embedded COF-based ultrathin stable hybrid composite materials could be an ideal solution for device-based (such as membranes) excellent dynamic sequestration of specific metal oxoanions with ultrafast kinetics and very selective efficiency (Scheme 1). Considering this, here we hypothesized that the utilization of a triaminoguanidine (TAG) moiety based ionic COF (iCOF) could be an excellent choice as an efficient host matrix for threading guest-MOPs through the hybridization strategy to fabricate multifunctionalized hybrid nanocomposites. This is particularly important owing to their thin 2D structure (exfoliated) and high stability and the presence of excess free counter anions, which can be exchanged with targeted TcO_4_^−^ oxoanions.

**Fig. 1:**
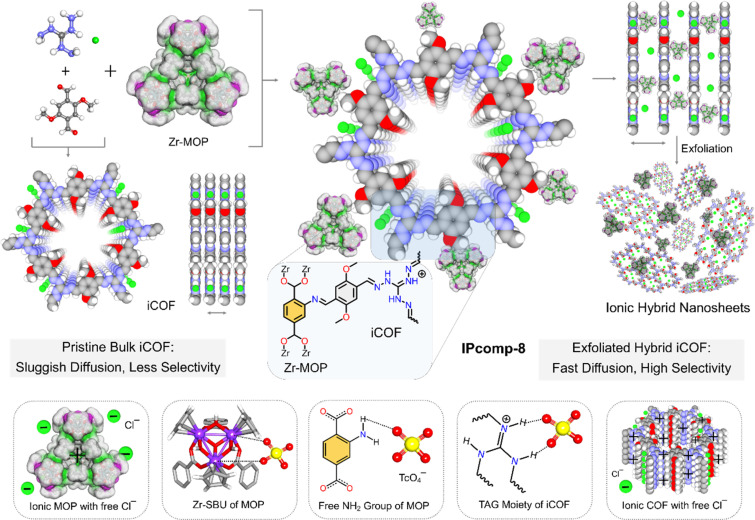
Schematic representation of synthesis of an ultrathin hybrid composite material (IPcomp-8) by covalently embedding amino group functionalized Zr-MOP with iCOF and pristine iCOF. Various multifunctional features of IPcomp-8 showing ion-exchange electrostatic and functional group mediated interactions with radioactive oxoanions (color code: gray: carbon, white: hydrogen, red: oxygen, green: chlorine, light blue: nitrogen, violet: zirconium).

Taking all the above facts into consideration, herein an efficient anion exchangeable ultrathin hybrid composite material (IPcomp-8, (where “IP” stands for IISER Pune and “comp” stands for composite)) has been synthesized through covalent grafting of amino-functionalized nanosized cationic Zr-MOP with a chemically stable positively charged COF (iCOF) (Fig. 1). We have strategically designed and grafted the NH_2_-functionalized cationic MOPs into the ionic COF matrix, where the excess of exchangeable Cl^−^ anions from both host-iCOF and guest-Zr-MOP, free –NH_2_ functional groups of the MOP, and the triaminoguanidine (TAG) moiety of the iCOF accelerate the anion exchange process due to the electrostatic and hydrogen-bonding interactions with the radioactive oxoanions (TcO_4_^−^) (Fig. 1). Most importantly, the Cp_3_Zr_3_O(OH)_3_ secondary building unit (SBU) of the MOP can selectively interact with TcO_4_^−^ through an exchange with its labile hydroxyl groups.^44–46^ Also, the iCOF matrix tightly harboured the MOP molecules keeping most of the active sites well exposed for favorable interactions. In addition to this, the covalent positioning of the cationic MOP into the cationic COF architecture results in easy and self-exfoliation into ultrathin hybrid nanosheets, owing to the strong interlayer ionic repulsion at play. This further allows full exposure of all the multifunctional active sites originating from both the Zr-MOP and iCOF matrix, further triggering rapid mass diffusion and high selectivity towards target specific TcO_4_^−^ anions in water. Therefore, IPcomp-8 was found to exhibit ultrafast and highly selective separation of radioactive TcO_4_^−^ or ReO_4_^−^ (surrogate) anions from the contaminated samples in the presence of large excesses (∼10 to ∼5000 fold) of other competing anions in both high (∼50 ppm) and ultralow (∼1000 ppb) concentrations. The composite also demonstrated TcO_4_^−^ entrapment from a wide range of pH and various irradiation doses with fast adsorption kinetics and the sorption was found to be reproducible. It could significantly lower the concentration of ReO_4_^−^ to well below the WHO-set permitted level for drinking water (10 ppb).^1^ Moreover, taking advantage of ultrathin nanosheets of the developed composite, we further fabricated a hybrid membrane, which demonstrated a highly efficient flow-through ReO_4_^−^ extraction from contaminated water. Such a swift and effective adsorption performance by cooperative multifunctionalities of IPcomp-8 was further supported by several experimental and theoretical calculation studies. Our findings point to a promising area for tackling issues with TcO_4_^−^ management and nuclear waste around the world.

## Results and discussion

**Fig. 2:**
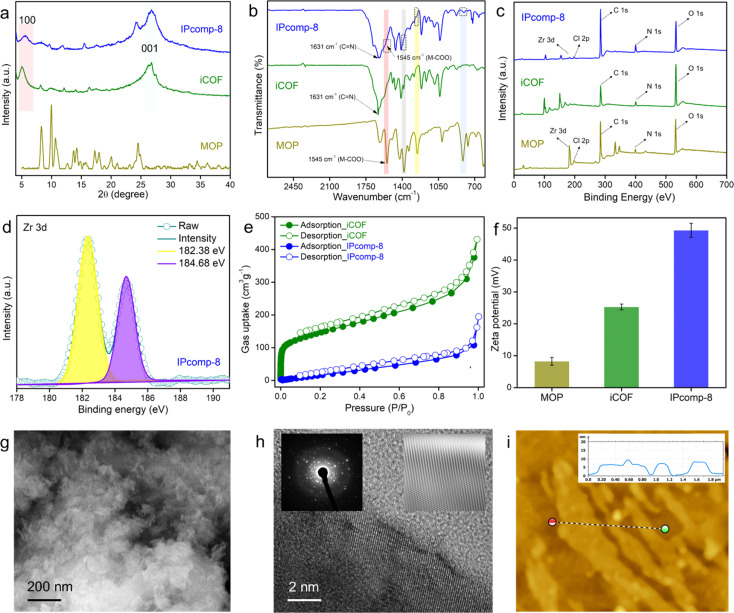
(a) PXRD profile, (b) FTIR spectra, and (c) XPS survey spectra of IPcomp-8 along with the pristine materials, (d) deconvoluted Zr 3d XPS spectra of IPcomp-8, (e) N_2_ sorption profile of iCOF and IPcomp-8 at 77 K, (f) comparison of zeta potential values of MOP, iCOF and IPcomp-8, (g) FESEM and (h) HRTEM images of IPcomp-8, (inset: lattice fringes and SAED images), and (i) AFM image and the corresponding height profile of IPcomp-8.

Initially, a discrete, chemically and hydrolytically stable, amino-group functionalized, nanosized (∼2 nm) cationic MOP ({[Cp_3_Zr_3_O(OH)_3_]_4_(NH_2_–BDC)_6_}·Cl_4_) was synthesized by reacting Zr(iv) metal with a 2-aminoterephthalic acid ligand and was characterized according to the previous literature report (Scheme S1 and Fig. S1–S5†).^47^ The triaminoguanidine (TAG) functionalized chemically robust cationic COF (iCOF) was also synthesized and thoroughly characterized following previously reported literature (Schemes S2, S3 and Fig. S6–S9†).^48^ After synthesizing these pristine host matrix (iCOF) and guest nanotraps (Zr-MOP), the cationic Zr-MOP/iCOF hybrid material (IPcomp-8) was fabricated *via* a multicomponent condensation reaction by reacting NH_2_-functionalized Zr-MOP with the precursors of iCOF by a solvothermal method (Fig. 1) (see the ESI† for a detailed synthetic procedure). The free amino groups of the MOP can serve as an extra precursor, to react with dimethoxyterephthalaldehyde and triaminoguanidine monomers of the iCOF to fabricate covalently linked hybrid composite porous materials (IPcomp-8). By varying the weight of the MOP content, a series of hybrid materials have been synthesized. After the synthesis, thorough characterization studies of the hybrid composite have been performed in order to explore its detailed structural insights. The structural analysis of the composite material was deduced through a combination of powder X-ray diffraction (PXRD), Fourier transform infrared spectroscopy (FT-IR), X-ray photoelectron spectroscopy (XPS), nitrogen gas sorption studies, field emission scanning electron microscopy (FESEM), transmission electron microscopy (TEM), atomic force microscopy (AFM), and others. The formation of the crystalline structure of the composite material was established from PXRD studies (Fig. 2a). The PXRD analysis of IPcomp-8 indicated identical diffraction peaks as the pristine iCOF, further validating the formation and retention of the crystalline structural integrity of the iCOF backbone in the composite.^48^ However, a decrease in intensity of the peak corresponding to the 100 plane and the broadening of the 001 plane peak have been observed. This could be due to the random displacement of the 2D layers of the iCOF matrix in IPcomp-8. As a result, the reflection corresponding to the 100 plane becomes weak. The broader peak at 2*θ* (∼25°) is due to the lack of π–π stacking between the iCOF layers of IPcomp-8, which is substantially influenced by the reduction of stacked layers in the composite.^49^ FT-IR analysis was utilized to investigate the formation of the composite between the guest NH_2_-MOP and host iCOF matrix. The FT-IR spectrum of IPcomp-8 indicated the appearance of a new prominent absorption band at ∼1631 cm^−1^ corresponding to C=N stretching (imine bonds) and disappearance of an absorption band at 1672 cm^−1^ corresponding to C=O stretching (aldehyde), as well as the presence of bands in low wavenumber regions, corresponding to metal–carboxylate bonds of the MOP, indicating a successful condensation reaction and grafting of Zr-MOPs into the iCOF matrix (Fig. 2b).^46–50^ Also, the bands of N–H stretching at ∼1439 and ∼3423 cm^−1^ confirm the presence of few free NH_2_ functional groups in the composite material (Fig. S10†).

The XPS survey spectra of the composite clearly indicated the presence of all relevant elements, including C, N, O, Zr, and Cl, as compared to pristine iCOF, validating the grafting of Zr-MOP in the porous structure of the iCOF (Fig. 2c). Moreover, the deconvoluted XPS spectra of the Zr 3d energy level of IPcomp-8 were found to be shifted towards lower binding energy compared to Zr 3d of the pristine MOP, indicating interaction between the guest MOP and host iCOF matrix (Fig. 2d and S11†).^51^ The thermogravimetric analysis (TGA) of IPcomp-8 illustrated an improved thermal stability of the MOP molecules when anchored into the iCOF matrix with no disintegration of the structure up to 400 °C (Fig. S12†). The presence of featured signals of both MOP and iCOF in the ^13^C solid-state CP-MAS NMR spectrum of the composite material denoted that IPcomp-8 consisted of both MOP and iCOF structures (Fig. S13†). Thereafter, the nitrogen gas sorption isotherm was recorded at low temperature (77 K) to assess the porous nature of the nanocomposite in comparison to the pristine material. The isotherm profile of IPcomp-8 revealed relatively less uptake of N_2_ gas at low relative pressure, showing that the composite is less porous. Also, the sorption data exhibited significantly lower gas uptake and thus lower surface area of IPcomp-8 than the pristine iCOF, which imply the successful incorporation of Zr-MOP inside the iCOF matrix as well as exfoliation into ultrathin nanosheets (Fig. 2e).^52^ Furthermore, in order to evaluate the ionic properties of IPcomp-8, zeta potential measurement has been performed. The surface charge of IPcomp-8 was found to be positive with a zeta potential value of +49.3 ± 2.1 mV, which inferred its high cationic nature (Fig. 2f). Importantly, the surface charge of the pristine cationic MOP and iCOF was found to be lower than that of the composite. The nanoscale level surface morphology of IPcomp-8 was examined by the FESEM, TEM and AFM analyses. The high-resolution FESEM images of IPcomp-8 showed robust sheet like morphology with plenty of small thin layers (Fig. 2g and S14†), while the pristine MOP and iCOF showed cube like microcrystalline and bulk-flake like morphology with micron to submicron layers, respectively (Fig. S14†).^48,51,52^ Similar thin sheet like morphology was found in the case of TEM analysis (Fig. S15†). The fast Fourier transformation (FFT) images from the high resolution TEM (HRTEM) experiment showed a highly crystalline nature with clear lattice fringes and a selected area electron diffraction (SAED) pattern of the IPcomp-8 structure (Fig. 2h(insets)).^53^ Additionally, the energy dispersive X-ray (EDX) examination of IPcomp-8 revealed a Zr/Cl ratio of ∼2.9 (in agreement with the crystallographic information of the Zr(iv)-MOP)^47^ confirming the existence of stable cationic MOP molecules in the iCOF matrix (Tables S1–S5†). The EDX and high-angle annular dark-field (HAADF) TEM elemental mapping of the composite materials from TEM experiments demonstrated the even distribution of the MOPs' constituent elements (Zr distribution) along with the other relevant elements (Fig. S16 and Tables S1–S5†). Moreover, the AFM experiment has been performed to explore the surface morphology of IPcomp-8 with greater details. The AFM images of IPcomp-8 demonstrated an ultrathin sheet like morphology with an average height of ∼4 to 7 nm (Fig. 2i). However, the AFM morphology of the pristine iCOF exhibited bulk sheets with an average height of ∼40 nm (Fig. S17†). These data clearly indicated the ultrathin morphology (nanosheets) of the hybrid composite. The formation of such an ultrathin structure of IPcomp-8 was mainly attributed to the exfoliation of the 2D layers of iCOF in the hybrid composite induced by intermolecular ionic repulsion between the adjacent iCOF layers, which is further originated due to the presence of charged (cationic) MOP molecules inside the iCOF structure as well as the cationic backbone of the iCOF matrix (Fig. 1).^52–55^ Moreover, the optical characteristics of the hybrid composite also helped understand the presence of luminescent amino-functionalized MOP within the iCOF matrix. The hybrid composite's UV-vis diffuse reflectance spectroscopy (DRS) spectra revealed distinct absorption peaks for both the MOP and iCOF, demonstrating the presence of both components in the hybrid composite (Fig. S18†). Apart from these characterization studies, detailed control experiments have been performed to investigate the stability and a leaching test of the covalently bound (through the imine bond formation between free amine groups of MOPs and the aldehyde group of iCOF, Fig. 1) MOP molecules with iCOF was conducted, which indicated successful formation of the Zr-MOP/iCOF hybrid composite materials (IPcomp-8) (see ESI notes 1 and 2, Fig. S19–S24†). Furthermore, the hybrid composite's stability to irradiation was studied by analysing gamma ray irradiated samples using PXRD and FT-IR analysis (Fig. S25 and S26†).

### Capture studies

**Fig. 3:**
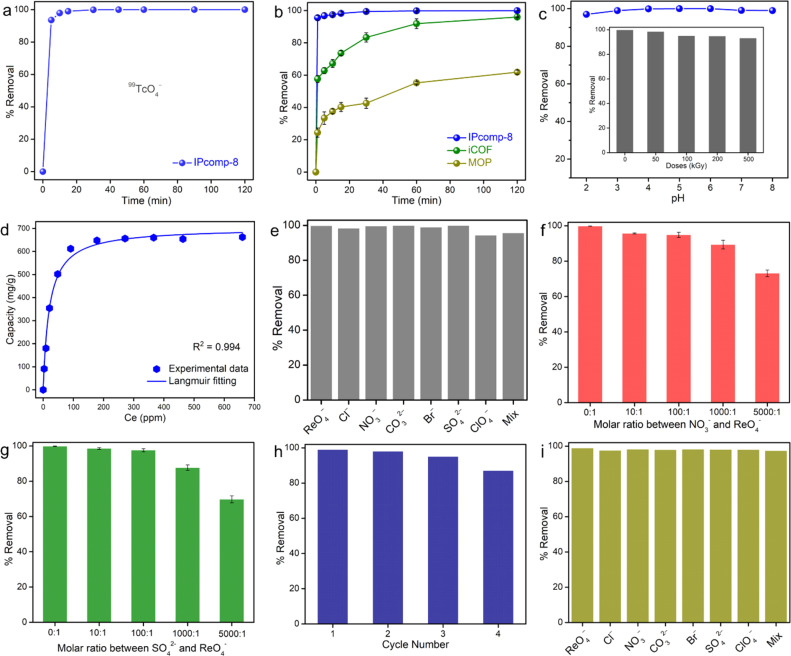
% Removal of (a) TcO_4_^−^ and (b) ReO_4_^−^ anions in different contact times by IPcomp-8, MOP and iCOF, conditions: [Re]_initial_ = 25 ppm and *m*_sorbent_/*V*_solution_ = 1 g L^−1^. (c) Removal efficiency of IPcomp-8 towards TcO_4_^−^ at different pH and (inset) different doses of γ-irradiation, and (d) Langmuir isotherm fitting model for ReO_4_^−^ capture, conditions: contact time = 24 hours and *m*_sorbent_/*V*_solution_ = 1 g L^−1^. (e) Result of selectivity for ReO_4_^−^ removal efficiency in the presence of different anions with high concentrations, conditions: [Re]_initial_ = 25 ppm, contact time = 24 hours, and *m*_sorbent_/*V*_solution_ = 1 g L. (f and g) Selectivity result for ReO_4_^−^ in the presence of excess NO_3_^−^ and SO_4_^2−^ anions, respectively, and (h) recycle test result of IPcomp-8 for ReO_4_^−^ ions, conditions: [Re]_initial_ = 25 ppm, contact time = 24 hours, and *m*_sorbent_/*V*_solution_ = 1 g L^−1^. (i) Selectivity of IPcomp-8 towards ReO_4_^−^ capture at an ultralow concentration, conditions: [Re]_initial_ = 1000 ppb, contact time = 24 hours, and *m*_sorbent_/*V*_solution_ = 1 g L^−1^.

IPcomp-8 displayed a stable colloidal suspension in water for long time with the characteristic Tyndall effect owing to its intrinsic ionic nature (Fig. S27†).^55^ The presence of excess free exchangeable Cl^−^ anions and multiple interaction sites, including the Zr(iv) secondary building unit (SBU) and amino functional group of the MOP and cationic TAG moiety of the iCOF in the hybrid composite material enthused us to examine its sequestration capability toward toxic/hazardous oxoanions in water.^56–58^ Metal derived radioactive oxoanion, TcO_4_^−^, and its surrogate oxoanion ReO_4_^−^ have been chosen for the sequestration study. Initially, to optimize the performance, we carried out the capture study with a series of compounds (composites synthesized with different amounts of MOPs) in ReO_4_^−^ solution (∼25 ppm) treated for 5 min. From the result of the inductively coupled plasma mass spectroscopy (ICP-MS) analysis it was found that the composite made by taking ∼15 mg of MOP (IPcomp-8) showed the best ReO_4_^−^ sequestration performance (Fig. S28†). Thereafter, all the following metal-oxoanion sequestration studies have been performed with IPcomp-8. Efficient extraction of radioactive metal pollutants from nuclear waste systems being a key-targeted criterion, at first, the effect of contact time of IPcomp-8 towards TcO_4_^−^ has been evaluated by batch adsorption experiments. The kinetics study of TcO_4_^−^ capture was conducted with the radiometry method where IPcomp-8 was found to exhibit ultrafast sequestration performance as within less than 5 min of contact time, almost >93% removal efficiency was observed (Fig. 3a). Notably, the fast sorption kinetics of IPcomp-8 is comparable to that of the other record holding state-of-the-art adsorbents and substantially faster than that of the majority of the reported sorbents. In this context, it is important to note that the majority of the widely used adsorbents for TcO_4_^−^ sorption, including commercial anion-exchange resins, Purolite A532E and A530E,^59^ cationic MOFs,^60–62^ inorganic materials,^11,63^*etc.*, were found to take a comparatively longer time to reach sorption equilibrium. This ultrafast exchange kinetic result of IPcomp-8 can help quickly respond to a nuclear incident and lower the risk posed by a ^99^TcO_4_^−^ leakage, which further encouraged us to carry out detailed TcO_4_^−^ sequestration studies. Now, owing to the high radioactive nature and low availability of ^99^TcO_4_^−^, the following high concentration batch anion-exchange experiments have been performed with nonradioactive surrogate ReO_4_^−^ owing to their identical chemical behaviour. For this, the time-dependent sequestration study was performed by taking IPcomp-8 in 25 ppm of stock ReO_4_^−^ solution. After a certain contact time interval, the compound was filtered and the residual concentration was measured. The result from ICP-MS data indicated the significant lowering of the Re(vii) concentration in very little time, as almost 95% ReO_4_^−^ removal efficiency was observed within less than 1 min in the case of IPcomp-8 (Fig. 3b, and S29†). Furthermore, the sorption kinetics towards ReO_4_^−^ was found to follow the pseudosecond order kinetics model with a high correlation coefficient of *R*^2^ > 0.999 for IPcomp-8. The rate constant value of the hybrid showed an ultrafast sorption rate than pristine materials (Fig. S28†). Inspired by this result as well as in order to evaluate the advancement of the hybrid composite (IPcomp-8) over the individual components towards improved TcO_4_^−^ sequestration, similar uptake study was conducted with the pristine MOP and iCOF. To our delight, in this comparison study, the composite material was found to demonstrate higher removal efficiency than both the bare MOP and iCOF (Fig. 3b, and S29†). Within less than 1 min of contact time, IPcomp-8 exhibited ∼95% removal efficiency, whereas MOP and iCOF exhibited significantly lower uptake efficiencies, at ∼25% and ∼57%, respectively. This outcome demonstrates the hybrid composite's superiority over distinct substances in metal oxoanion separation applications (Fig. S30–S32†).

The pH of the nuclear wastewater or industrial wastewater system may vary from acidic to alkaline depending upon the environment of the discharge system. Therefore, it is important to check the sequestration efficiency of a sorbent material in a wide pH range. Interestingly, IPcomp-8 was found to eliminate TcO_4_^−^ across a wide range of pH without losing its actual efficiency (Fig. 3c). In addition, considering the strong ionizing field in nuclear waste remediation, the sequestration of TcO_4_^−^ by IPcomp-8 was evaluated after irradiation with high doses of γ-irradiation. To our delight, the hybrid exhibited excellent elimination of TcO_4_^−^ even after exposing γ-irradiation, further indicating high radiation tolerance ability (Fig. 3c(inset)). Next, on account of the enormous inventory of ^99^TcO_4_^−^ in used nuclear waste, the adsorption capacity of an adsorbent is a fundamental parameter in order to understand its full efficiency. The saturation capacity of IPcomp-8 for ReO_4_^−^ was estimated by carrying out the adsorption isotherm experiment. In a typical test, a range of initial concentrations (20–800 ppm) of ReO_4_^−^ aqueous solution was treated with IPcomp-8 for a contact period of 24 h to reach sorption equilibrium. The uptake of ReO_4_^−^ by IPcomp-8 was found to follow the Langmuir isotherm model with the high correlation coefficient value, which indicated the monolayer sorption mechanism (Fig. 3d). When the sorption capacities were plotted as a function of equilibrium concentration, the maximum sorption capacity of ReO_4_^−^ by IPcomp-8 was found to be ∼632 mg g^−1^. However, a reduction in the capacity was noticed when measuring the maximum adsorption capacity of the IPcomp-8 sample after γ-ray irradiation towards high concentrations of ReO_4_^−^ (Table S6†). It should be mentioned that apart from the kinetics and capacity, selectivity of an adsorbent is another highly important parameter to realize its potential towards real-time application. Therefore, the efficacy of an adsorbent towards a specific analyte in the presence of an excess of other interfering analytes should be tested. According to earlier literature, it has been observed that the majority of studies have mainly focused on uptake capacity, but very little attention has been paid to the selectivity aspect in the presence of other interfering analyte anions, which is crucial for sequestration from the actual wastewater system.^19,62–65^ Nuclear or industrial wastewater contains anions such as Cl^−^, NO_3_^−^, Br^−^, SO_4_^2−^, ClO_4_^−^, *etc.*, in enormous excess amounts, which interfere in the adsorption process.^57–62^ Initially, to check the influence of these aforementioned interfering anions in this adsorption process, a similar ReO_4_^−^ capture experiment has been performed by taking a binary mixture of ReO_4_^−^ as the target anion along with these anions individually in a similar higher concentration. To our delight, it was found that the ReO_4_^−^ sorption efficiency by IPcomp-8 remained intact (almost 98%) in the presence of other competing anions (Fig. 3e). Inspired by this result, we were motivated further to test the efficiency of IPcomp-8 towards ReO_4_^−^ in the presence of surplus concentrations (∼10, ∼100, ∼1000, and ∼5000-fold) of two specific interfering analytes such as NO_3_^−^ and SO_4_^2−^, which are generally omnipresent with TcO_4_^−^ or ReO_4_^−^ in wastewater. By performing a similar sorption test, we observed no significant effect of both NO_3_^−^ and SO_4_^2−^ competing ions on the sequestration efficiency of IPcomp-8 up to a ∼10-fold concentration (Fig. 3f and g). Even though the concentration of NO_3_^−^/SO_4_^2−^ is in a ∼100-fold excess, IPcomp-8 exhibited as high as >95% removal percentage towards ReO_4_^−^. In the case of excessive concentrations (∼1000 and ∼5000-fold), the ReO_4_^−^ removal efficiency of IPcomp-8 was found to diminish (Fig. 3f and g). However, IPcomp-8 was still found to exhibit ∼89% and ∼74% ReO_4_^−^ removal efficiency in the presence of ∼1000 and ∼5000-fold NO_3_^−^, respectively (Fig. 3f). In addition to this, the composite also demonstrated superior removal efficiency towards ReO_4_^−^ in the presence of SO_4_^2−^ anions, which is highly competitive owing to its high charge/size ratio (Fig. 3g). The distribution coefficient value (*K*_d_) of an adsorbent is an important parameter, which is used to explore its binding affinity towards a specific analyte.^66^ The *K*_d_ values of IPcomp-8 for ReO_4_^−^ in the presence of these aforementioned competing anions were further calculated, which exhibited as high as in 10^6^ order for all cases (Table S7†). All these results validate the highly selective nature of IPcomp-8 towards TcO_4_^−^ radioactive anions, making it a suitable adsorbent with potential for practical application. Such excellent selectivity of IPcomp-8 for TcO_4_^−^ or ReO_4_^−^ originates from the multifunctional properties of the composite, such as Zr(iv)-SBU, free NH_2_-groups and TAG moieties. Next, to be an ideal adsorbent, recyclability is another imperative requirement. The hybrid material loaded with ReO_4_^−^ anions was regenerated using saturated Na_2_SO_4_ solution by stirring it for ∼10 hours. Even after four cycles, it was discovered that IPcomp-8 could be easily recycled without significantly losing its sorption effectiveness, demonstrating exceptional regeneration capability (Fig. 3h).

Overall, the IPcomp-8 hybrid material is superior to conventional adsorbents due to its rapid capture efficiency with high adsorption capacity and unparalleled selectivity toward TcO_4_^−^ or ReO_4_^−^. Due to accidental discharge in a real water body the concentration of the toxic analyte exists in a very low concentration (<1 ppm).^1^ Therefore, it is important to explore the ability of the adsorbent to sequestrate the target analyte at trace concentrations. By virtue of the rapid and efficient TcO_4_^−^ or ReO_4_^−^ oxoanion adsorbing behaviour of IPcomp-8, treatment of ReO_4_^−^ contaminated wastewater was performed, by mimicking the real time scenario. Moreover, an ultralow-concentration (∼1000 ppb) ReO_4_^−^ sequestration study in the presence of an abundance of other competing ions was also carried out and monitored using ICP-MS analysis to show the material's effectiveness. Initially, in the kinetics study, IPcomp-8 was found to exhibit ultrafast sequestration efficiency towards ReO_4_^−^ achieving ∼99% removal efficiency within less than 1 min (Fig. S33†). Consequently, the concentration of ReO_4_^−^ was found to decrease rapidly, and the final concentration was found to be much below the WHO permitted level of drinking water (Fig. S34†).^67^ Furthermore, the kinetic study was found to follow pseudo-second order kinetics with a high rate constant value (Fig. S35†). Moreover, the selectivity of IPcomp-8 in low concentrations was also investigated taking all the aforementioned coexisting anions. From the selectivity test, it was found that IPcomp-8 exhibits superior ReO_4_^−^ capture efficiency even in the presence of all other anions in equal as well as highly excess concentrations (Fig. 3i, S36 and S37†). All these results of efficient and selective entrapment of radioactive TcO_4_^−^ oxoanions indicated the potential of IPcomp-8 towards real-time nuclear wastewater treatment application.

### Adsorption mechanism studies

**Fig. 4:**
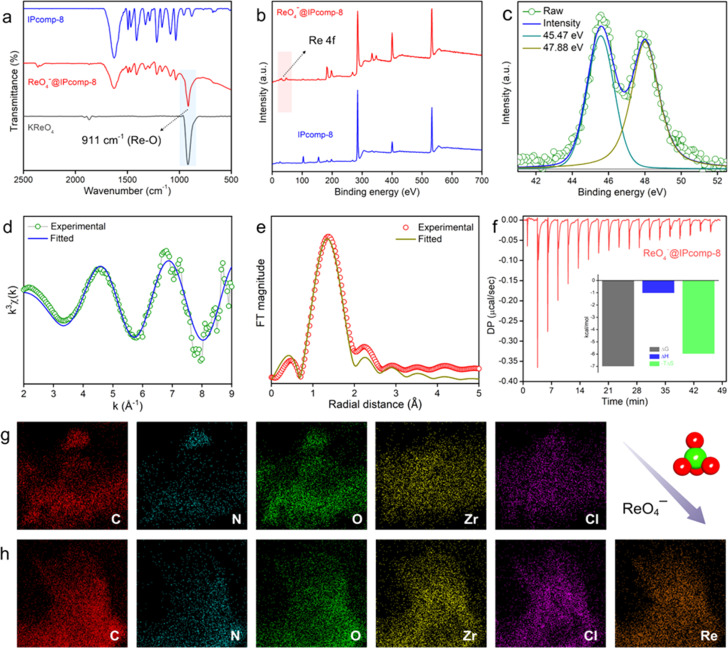
(a) FT-IR spectra of ReO_4_^−^ loaded IPcomp-8, (b) XPS survey spectra and (c) deconvoluted Re 4f core-level XPS spectra of IPcomp-8 and ReO_4_^−^@IPcomp-8, (d) raw and (e) Fourier transforms of Re L3 edge EXAFS spectra of ReO_4_^−^ treated IPcomp-8, (f) ITC thermogram with its corresponding parameters, and (g and h) TEM EDX elemental mapping of IPcomp-8 and ReO_4_^−^@IPcomp-8, respectively.

After evaluating the capture performance, the underlying sorption mechanistic insight was further explored experimentally and theoretically in order to understand the multiple interactions that are responsible for such efficient TcO_4_^−^ sequestration performance by IPcomp-8. For this, the following post capture characterization studies have been performed. At first the FTIR analysis of ReO_4_^−^ loaded IPcomp-8 displayed the appearance of a sharp peak at ∼911 cm^−1^ corresponding to Re–O asymmetric stretching of ReO_4_^−^ anions, which supports the binding of ReO_4_^−^ with IPcomp-8 (Fig. 4a).^57–62^ The thermogravimetric analysis of the ReO_4_^−^ loaded material showed similar thermal stability compared to the pristine IPcomp-8 (Fig. S38†). Thereafter, the XPS analysis was performed to understand the nature of interaction between the positively charged backbone of the composite material and the ReO_4_^−^ oxoanion. The Re 4f binding energy peak was clearly visible in the XPS survey spectra of samples that had undergone ReO_4_^−^ treatment, indicating that Re was present on the surface of IPcomp-8 (Fig. 4b). The deconvoluted XPS spectra of the Re 4f core-level confirmed the existence of Re in their +7 oxidation state (ReO_4_^−^) (Fig. 4c).^57–62^ Furthermore, extended X-ray adsorption fine structure (EXAFS) study was performed in order to understand the local structure around the Re atoms in ReO_4_^−^ loaded IPcomp-8. The fitting analysis of the raw data and Fourier transforms of Re L3 edge K^3^-weighted spectra of ReO_4_^−^ loaded IPcomp-8 indicated a Re–O shell with a radial distance (*R*) of 1.734 Å and the coordination number of Re–O was found to be 4 (Fig. 4d and e). These values were found to be close to the matric parameters of the referenced material (Table S8 and Fig. S39†).^68^ A further detailed EXAFS analysis of the Zr metal center of the ReO_4_^−^ treated IPcomp-8 supported the stable existence of the MOP within the iCOF matrix of the composite after the capture test (ESI note 3, Table S9 and Fig. S40–S42†). Importantly, it was discovered that the EXAFS results and the XPS data correlated, confirming that Re species maintain their +7 oxidation states and that the adsorption process does not include Re(vii) reduction.^69^ This finding demonstrated unequivocally that the strong electrostatic interactions between the cationic structure of IPcomp-8 and ReO_4_^−^ were responsible for the excellent removal efficiency.

**Fig. 5:**
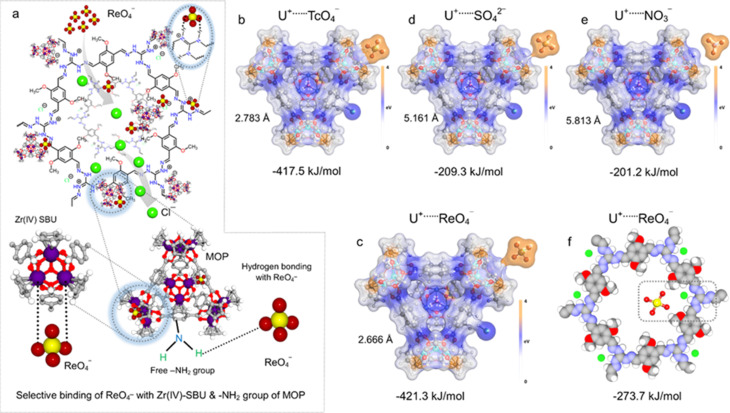
(a) Illustration of mechanistic insight of selective ReO_4_^−^ capture through different interactions by IPcomp-8. Density functional theory (DFT) calculations: (b–f) energy-optimized adsorption complexes for interactions between TcO_4_^−^ or ReO_4_^−^ with Zr(iv)-SBU of the MOP and iCOF structural units with their corresponding binding energies.

Next, we have also performed an isothermal titration calorimetry (ITC) experiment to evaluate thermodynamic feasibility of the favourable interactions between the compound and the analyte in an aqueous medium (ESI note 4†). The ITC experiment quantitatively provides the change in enthalpy, entropy and Gibbs free energy associated with the adsorption driven by the ion-exchange process between the adsorbent and adsorbate.^70^ The recorded thermogram depicted a high association constant with an exothermic binding event along with the parameter. This result indicated that the adsorption of ReO_4_^−^ by IPcomp-8 was favourable as the Gibbs free energy of the process was found to be negative with a positive association constant and positive entropy (Fig. 4f and S43†). Thus, the ITC data clearly demonstrated the similarities between the thermodynamic parameters and the experimental findings of strong interaction of IPcomp-8 and ReO_4_^−^. Furthermore, the HRTEM image of ReO_4_^−^ treated IPcomp-8 showed identical morphological configuration with retained crystalline nature (Fig. S44†). This further explored the robust nature of the hybrid composite material towards wastewater treatment. The EDX elemental mapping analysis from the TEM experiment of ReO_4_^−^@IPcomp-8 was performed, which indicated the appearance of homogeneous distribution of Re metal ions along with other relevant elements throughout the structure of the composite (Fig. 4g, h, and S45†). All these above investigations and characterization studies were carried out to support the possible ReO_4_^−^ or TcO_4_^−^ binding mechanism with IPcomp-8. Previously in the literature it was well established that Zr(iv) SBU and amino functional groups can act as active sites for selective oxoanion binding.^46,71^ As a result, the composite material's cationic guest (NH_2_-MOPs) has a significant impact on the selective trapping of TcO_4_^−^. Having said that, pristine MOP molecules undergo self-aggregation,^41^ which inhibits the functional groups from interacting with the incoming oxoanions; hence this results in the reduction of the capture efficiency. In the IPcomp-8 hybrid composite material nanosized MOPs are covalently tethered with the layers of the iCOF that restricts the self-aggregation process and consequently exposed more number of active binding sites for selective interaction with metal oxoanions. Moreover, when the MOP molecules bind to the iCOF it is exfoliated driven by electrostatic repulsions and forms ultrathin hybrid nanosheets. These nanosheets bear a greater number of exposed interactive sites with excessive free exchangeable Cl^−^ ions for the amplified strong electrostatic interactions between the cationic host matrix and anionic metal based oxoanions (ReO_4_^−^ or TcO_4_^−^) which enables ultrafast and selective capture efficiency. Additionally, the TAG functional moiety of the selected chemically stable iCOF network synergistically helped with the selective interaction with targeted metal oxo pollutants. IPcomp-8 exhibited highly efficient selective removal of ReO_4_^−^ oxoanions, from the contaminated water system. The multifunctional nature of the hybrid ultrathin nanosheet composite material can be rationalized to the efficient sequestration performance. Among them, the ultrathin nanosheet structure of the composite aids in the rapid diffusion of oxoanions through its exfoliated open porous structure towards the main active sites: guest-amino functionalized Zr(iv)-SBU-based cationic MOPs (Fig. 5a). Thereafter, the oxoanion TcO_4_^−^/ReO_4_^−^ interacts with the Zr-SBU and the free –NH_2_ group of the cationic MOP, followed by exchange with Cl^−^ ions of both the guest (MOP) and host matrix (iCOF). Such interactions can be attributed to the dispersive interactions or supramolecular interactions or dipole interactions of the TcO_4_^−^/ReO_4_^−^ with the functional groups of MOP molecules as well as the backbone of the iCOF matrix of the composite material. These selective strong interactions were further validated using theoretical calculations using a density functional theory (DFT) study, which quantitatively supported the experimental values. The interactions between the cationic MOP and fragment of iCOF (both abbreviated as U^+^) in IPcomp-8 and anions such as TcO_4_^−^ or ReO_4_^−^, SO_4_^2−^, NO_3_^−^, and others were investigated. The calculations showed that the Zr(iv)-SBU of the MOPs selectively interacts with TcO_4_^−^ and ReO_4_^−^ over other anions with their stable adsorption structure at binding energies of −417.5 and −421.3 kJ mol^−1^, respectively (Fig. 5b and c). The binding energies for other anions were less than those of TcO_4_^−^ and ReO_4_^−^ (Fig. 5d, e and S46†). Other bindings through a hydroxyl group (OH^−^), hydrogen of the –Cp ring and the amino functional group (–NH_2_) of the MOP are provided in the ESI file (Fig. S47 and S48).† In addition, the cationic backbone of the iCOF structure interacts with ReO_4_^−^ anions through hydrogen bonding with the TAG moiety at a binding energy of −273.7 kJ mol^−1^ (Fig. 5f). Such a difference between the binding energies of U^+^⋯TcO_4_^−^ or U^+^⋯ReO_4_^−^ with U^+^⋯other anions caused highly selective adsorption of TcO_4_^−^ or ReO_4_^−^ over other anions (such as NO_3_^−^, SO_4_^2−^, Cl^−^, Br^−^, *etc.*) through a robust ion-exchange mechanism.

### Membrane based continuous flow-through sequestration

**Fig. 6:**
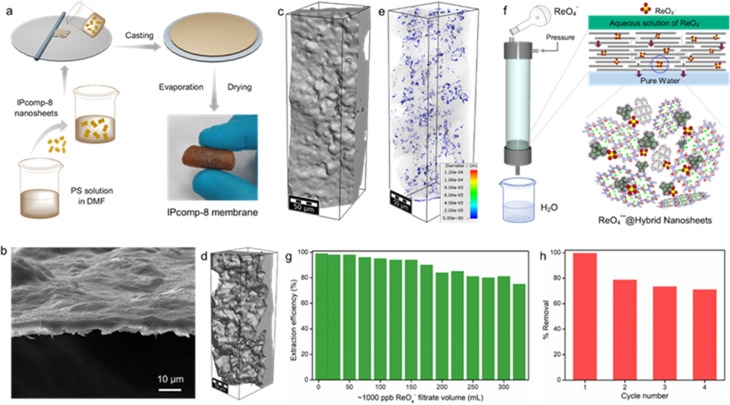
(a) Fabrication procedure and digital image of the IPcomp-8 based hybrid membrane. (b) SEM image, (c and d) volume rendered 3D X-ray tomography images of the IPcomp-8 based membrane. (e) Visualization of pore distribution with a colour-scale CT image. (f) Schematic representation of flow-through selective extraction of ReO_4_^−^ oxoanions by the IPcomp-8 based hybrid membrane. (g) Extraction efficiency of IPcomp-8 towards ReO_4_^−^ with different amounts of feed volume. (h) Regeneration result of the membrane, conditions: [Re]_initial_ = 1000 ppb, pH = 6.9.

Inspired by such excellent radioanion sequestration efficiency as well as the robust ultrathin 2D nanosheet nature of the hybrid COF composite material (IPcomp-8), we further sought to explore the potential of the material for large-scale continuous flow-through based TcO_4_^−^ or ReO_4_^−^ extraction by developing a hybrid membrane. At first an IPcomp-8 based stable hybrid membrane was fabricated by a typical mixed-matrix-membrane technique (Fig. 6a) (see Section S9.1†).^53^ The thus developed hybrid membrane was found to possess a smooth surface and compact packing without significant defects on the surface, as revealed from the SEM images of the membrane (Fig. 6b and S49†). In addition, the 2D cross-sectional images from the microscale X-ray computed tomography (CT) experiment indicated a similar smooth surface morphology of the membrane (Fig. S50†). Moreover, the volume rendered 3D CT images and its corresponding color-coded images of the membrane showed distribution of pores throughout the undulated surfaces on one side and relatively smoother ones on the other side (Fig. 6c–e and S51†). Task-specific applications such as sequestration of specific elements/molecules/ions through membranes hold great promise towards industrial application.^72^ Therefore, the extraction performance of the IPcomp-8 membrane for ReO_4_^−^ removal from contaminated samples was first evaluated (see Section S9.2†).

In a typical experiment, a ∼1000 ppb ReO_4_^−^ feed was charged through the hybrid membrane in a dynamic mode and the concentration of the membrane passed solution was measured by ICP-MS (Fig. S52†). As shown in Fig. 6f, after the treatment by a flow-through dynamic process the membrane was found to reduce the concentration of the ReO_4_^−^ solution to below ∼10 ppb. As a result of the adsorption-based membrane separation, the IPcomp-8 membrane was found to exhibit more than ∼93% removal efficiency up to 170 mL of ReO_4_^−^ feed solution (Fig. 6g). After that, the removal efficiency started to decrease and was observed to be intact as >70% up to 315 mL of contaminated solution. However, after that, the removal efficiency of the membrane was significantly decreased because of blocking of most of the active sites through binding with ReO_4_^−^. Moreover, the recycling ability of the membrane was further investigated using saturated Na_2_SO_4_ solution, which greatly regenerated the capture performance of the hybrid membrane. In the recycling test, the membrane was found to demonstrate more than ∼75% removal efficiency up to 250 mL of feed ReO_4_^−^ solution in all four cycles (Fig. 6h). From the perspective of practical application, this study demonstrated the exceptional segregation effectiveness of the IPcomp-8 membrane towards TcO_4_^−^ oxoanions in both environmental and industrially contaminated water. The cooperative impact of the exposed ultrathin hybrid nanosheets, in addition to the presence of Zr-SBU, free –NH_2_ groups, and exchangeable Cl^−^ ions in the overall nanocomposite, is thought to be responsible for the IPcomp-8 membrane's excellent selective separation performance.

## Conclusions

In conclusion, we have strategically designed and successfully synthesized a covalently linked MOP@iCOF cationic hybrid composite material (IPcomp-8), which demonstrated ultrafast and selective sequestration of TcO_4_^−^ oxoanions in water. This work demonstrates an optimization of a rationally designed strategy to remarkably enhance the sequestration kinetics and selectivity towards pertechnetate segregation from wastewater systems. Structural and optical characterization studies have been thoroughly carried out to gain detailed insights into the novel composite material. The major targeted criterion being the selective entrapment of TcO_4_^−^, careful anchoring of nanosized discrete cationic MOP molecules based on a Cp_3_Zr_3_O(OH)_3_ SBU into the chemically stable charged iCOF matrix produced unprecedented selectivity and ultrafast kinetics for TcO_4_^−^/ReO_4_^−^ capture. Notably, in the dynamic sorption technique, the membrane form of the hybrid composite illustrated unparalleled absorption ability toward TcO_4_^−^/ReO_4_^−^ from wastewater samples. This newly developed hybrid cationic material may be a possible contender for wastewater treatment due to its rapid uptake capability, excellent selectivity, and satisfactory recyclability. We believe that the current discoveries will pave the way for the creation of a range of efficient innovative sorbent materials for use in other environmentally important separation applications.

## Author contributions

W. M. conceived and designed the project. W. M., S. F. and D. M. conducted all the experiments and analyzed all the data. W. M. wrote the manuscript, while S. F., S. L., D. M. helped finalize the draft. S. F. designed the graphical art in the figures. A. S. performed all the radioactive studies. R. R. U. did the EXAFS measurement and fitting analysis. A. T. performed the tomography experiment. M. M. S. performed the theoretical studies. S. K. G. supervised the project. All authors have given approval to the final version of the manuscript.

## Conflicts of interest

The authors declare no competing interests.

## Supplementary Material

SC-015-D4SC04010D-s001

## Data Availability

All data used for this study are available in the paper and ESI files.†
