# Supplementary material for: A nanotrap infused ultrathin hybrid composite material for rapid and highly selective entrapment of 99TcO4−
Source: Chem Sci. 2024 Oct 8;15(44):18463–75. doi: 10.1039/d4sc04010d (PMC11485004; doi:10.1039/d4sc04010d)
Supplement: SC-015-D4SC04010D-s001 [file SC-015-D4SC04010D-s001.pdf]

Supplementary Information (SI) for Chemical Science.
This journal is © The Royal Society of Chemistry 2024

Supporting information file

Nanotrap Infused Ultrathin Hybrid Composite Material for Rapid and Highly Selective Entrapment of $^{99}TcO_4^-$

Writakshi Mandal,[a] Sahel Fajal,[a] Dipanjan Majumder,[a] Arijit Sengupta,[b,c] Sumanta Let,[a] Rajashri R. Urkude,[d,e] Mandar M. Shirolkar,[f,g] Arun Torris,[h] Sujit K. Ghosh*†[a,i]

[a]Department of Chemistry, Indian Institute of Science Education and Research (IISER) Pune, Dr. Homi Bhaba Road, Pashan, Pune 411 008, India.

[b]Radiochemistry Division, Bhabha Atomic Research Centre, Mumbai 400085, India.

[c]Homi Bhabha National Institute, Mumbai 400094, India.

[d]Beamline Development and Application Section Bhabha Atomic Research Centre, Mumbai – 400085.

[e]Accelerator Physics & Synchrotrons Utilization Division, Raja Ramanna Centre for Advanced Technology, Indore-452013.

[f]Advanced Bio-Agro Tech Pvt. Ltd, Baner, Pune 411045, India.

[g]Norel Nutrient Bio-Agro Tech Pvt. Ltd, Baner 411045 India.

[h]Polymer Science and Engineering Division, CSIR-National Chemical Laboratory, Dr. Homi Bhabha Road, Pune 411008, India.

[i]Centre for Water Research (CWR), Indian Institute of Science Education and Research (IISER) Pune, Dr. Homi Bhabha Road, Pashan, Pune 411 008, India.

Table of Contents

Section S1

Materials, General Characterizations and Physical Measurements. S3-S5

Section S2

Detailed procedures for synthetic preparation. S6-S7

Section S3

Oxoanion capture studies. S8-S11

Section S4

Structural characterization of synthesized materials. S12-S24

Section S5

Stability and Leaching test. S25-S32

Section S6

Capture studies. S33-S39

Section S7

Adsorption mechanism studies. S40-S47

Section S8

Theoretical calculations studies. S48-S50

Section S9

Mixed Matrix Membrane based studies S51-S54

Section S10

References S55-S55

Section S1: Materials, General Characterizations and Physical Measurements.

Materials: All the starting materials, reagents and solvents were purchased from Sigma-Aldrich, Alfa Aesar, TCI chemicals, Avra chemicals, and used as received.

General Characterizations and Physical Measurements:

Powder X-ray diffraction (PXRD): Powder X-ray diffraction (PXRD) experiments were performed on a Bruker D8 Advanced X-ray diffractometer at room temperature using Cu Kα radiation (λ = 1.5406 Å) at a scan speed of 0.5 ° min^{-1} and a step size of 0.01° in 2θ.

Fourier transform infrared spectroscopy (FT-IR): The IR Spectra were acquired by using a Bruker Optics ALPHA-E spectrometer with a universal Zn-Se ATR (attenuated total reflection) accessory. FT-IR data are reported with a wave number (cm^{-1}) scale in 500-4000 cm^{-1} range.

Thermogravimetric analysis (TGA): Thermogravimetric analyses were recorded on Perkin-Elmer STA 6000 TGA analyzer by heating the samples from 30 °C to 800 °C under N_2 atmospherewith a heating rate of 10 °C min^{-1}.

Scanning electron microscopy (SEM): The morphology of the crystalline materials was recorded with a Zeiss Ultra Plus field-emission scanning electron microscope (FESEM) with an integral charge compensator and embedded EsB and AsB detectors (Oxford X-max instruments 80 mm^2 (Carl Zeiss NTS, GmbH). The samples were sputter-coated with a 5-10 nm Au film to reduce charging. The elemental analysis was carried out using voltage of 15 KV equipped with an EDX detector. Data acquisition was performed with an accumulation time of 600s.

Transmission electron microscopy (TEM): TEM imaging and STEM-EDS were performed on the HRTEM (JEM-2200FS, JEOL) operating at an acceleration voltage of 200 kV. For TEM analysis, all the samples were dispersed in isopropanol and sonicated. Then, the samples were left for 2 min, and the upper part of the solution was taken for preparing TEM samples on a lacey carbon coated copper grid (Electron Microscopy Science, 200 mesh).

Nitrogen adsorption-desorption isotherm measurements: N_2 gas adsorption measurements were performed using BelSorp-Max instrument (Bel Japan). Prior to adsorption measurements, the activated samples were heated at 120 °C under vacuum for 12 hours using BelPrepvacII.

X-ray photoelectron spectroscopy (XPS): As-obtained powder samples were stuck to conductive paste and then measured by X-ray photoelectron spectroscopy using K-Alpha+model (Thermo Fischer Scientific, UK) with Al Kα source.

Nuclear magnetic resonance (NMR): ^{1}H and ^{13}C NMR spectra were recorded on Bruker 400 MHz NMR spectrometer. The chemical shifts are expressed in parts per million (δ scale).

High-resolution mass spectroscopy (HRMS): The mass analysis of the MOPs was carried out using high-resolution mass spectrometry (HRMS-ESI-Q-TOF LC-MS) and Applied Bio system 4800 PLUS matrix-assisted laser desorption/ionization (MALDI) TOF/TOF analyzer.

UV-visible absorption spectra: UV-vis absorption studies were performed on a Shimadzu UV 3600 UV /vis /NIR spectrophotometer in an optical quartz cuvette (10 mm path length) over the entire range of 200-800 nm.

Steady state photoluminescence spectra: The steady-state photoluminescence studies were recorded on a Fluorolog-3 spectrofluorometer (HORIBA Scientific).

Inductively coupled plasma atomic absorption/emission spectrometry (ICP-AAS/AES) and mass spectrometry (ICP-MS): ICP-AAS analysis was performed on Thermo Scientific iCE 3000 Series. ICP-AES analysis was performed on ARCOS, Simultaneous ICP Spectrometer. ICP-MS was performed on Quadrupole inductively coupled plasma mass spectrometry (Q-ICP MS; Thermo Scientific iCAP Q) instrument. Multielement standards were purchased from inorganic ventures.

Isothermal Titration Calorimetry (ITC): The ITC experiments were performed on a Malvern MicroCal PEAQ-ITC microcalorimeter having 20 (19+1) injection cycle method capacity, thevsample volume of 280 µL with a sample cell size of 200 µL and injection syringe volume of 40 µL for each set of experiments.

3D X-ray tomography: Hybrid aerogels were imaged using X-ray microtomography (Xradia 610 Versa X-ray Microscope, Zeiss X-ray Microscopy, Pleasanton, CA, USA) to study its morphology, porosity and pore-size distribution. Specimens were loaded onto the sample holder, kept in between the X-ray source and the detector assembly. Detector assembly

consisted of a scintillator, 20X optics and a CCD camera. X-ray source was ramped up to 80 kV and 7 W. The tomographic image acquisitions were completed by acquiring 5001 projections over 360° of rotation with a pixel size of 0.80 microns for a sample size of 0.8 x 0.8 x 0.8 mm^3. Each projection was recorded with 3 seconds of exposure time. In addition, projections without the samples in the beam (reference images) were also collected and averaged. The filtered back-projection algorithm was used for the reconstruction of the projections to generate two-dimensional (2D) virtual cross-sections of the specimens. Image de-noising, filtration, segmentation and further processing were performed using GeoDict software package (GeoDict® 2018, Math2Market GmBH, Germany). 2D images were trimmed down to a sub-volume (100 x 100 x 100 voxels with 0.80 cubic micron per voxel), filtered to remove noise and segmented after OTSU threshold selection based on local minima from the grayscale histogram. Resultant 3D reconstructed model was used to estimate the pore characteristics such as porosity, pore-size distribution, etc. using PoroDict® software package (GeoDict® 2018, Math2Market GmBH, Germany), where pore-radius is determined by fitting spheres into the pore volume.

Density Functional Theory (DFT) Simulation

The DFT simulation studies were carried out using Discovery Studio 2017 (BIOVIA, Dassault Systèmes). The calculations were performed using DMOL3 and B3LYP hybrid functions. The calculations were performed for the Determination of electrostatic-potential surface (ESP), binding sites and binding energy of interactions.

Section S2: Detailed procedures for synthetic preparation.

S2.1 Synthesis of Metal-organic Polyhedra (MOP): The MOP was synthesized accordance with literature report with slight modification.[1] 2-aminoterephthalic acid (50 mg, 0.3 mM) and Zirconocene dichloride (150 mg, 0.5 mM) were dissolved in N, N-dimethylacetamide (DMA, 10 mL) with a trace amount of water (40 drops) and kept it at room temperature and left undisturbed state for 72 hours. The yellow cubic block crystals were collected by filtration and dried under vacuum.

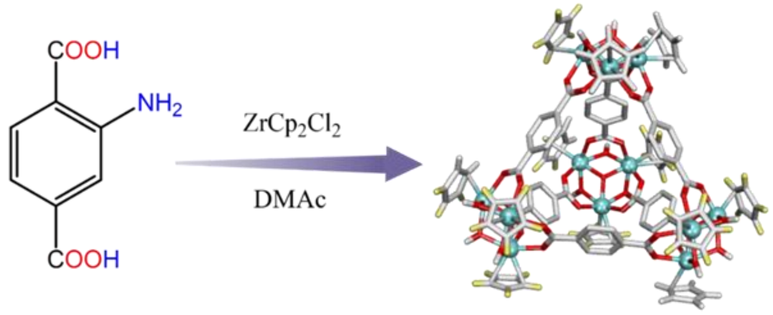

Scheme S1: Synthesis scheme of Zr(IV)-based metal-organic polyhedra (MOP).

S2.2 Synthesis of triaminoguanidine hydrochloride (TG_{Cl}): Triaminoguanidine hydrochloride (TG_{Cl}) was synthesized according to the reported protocol with some modifications.[2] First, guanidium hydrochloride was suspended in 1,4-dioxane (260 mL). After the addition of hydrazine monohydrate (86.6 mL, 1.782 mol), the mixture was heated at 110 °C for 2 hours. After cooling to room temperature, the reaction solution was filtered and continuously washed with 1,4-dioxane. The resulting white precipitate was dried under a high vacuum environment to obtain TG_{Cl}.

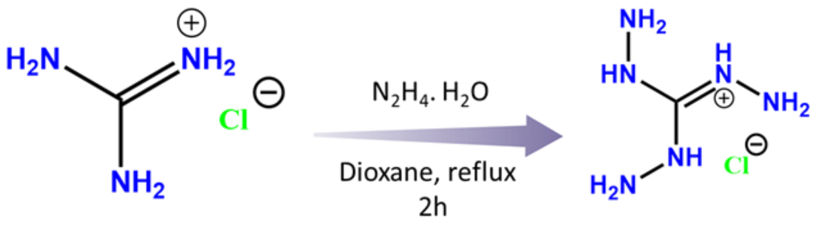

Scheme S2: Synthesis scheme of triaminoguanidine hydrochloride (TG_{Cl}) monomer.

S2.3 Synthesis of iCOF: The iCOF was synthesized accordance with literature report with slight modification.[2] A Pyrex tube was charged with TG_{Cl} (14.8 mg, 0.11 mM) and 2,5-dimethoxyterephthalaldehyde (29.1 mg, 0.15 mM) in a solution of deionized water/mesitylene/EtOH/3 M acetic acid (0.3/0.1/0.5/0.2 mL). The tube was flash frozen by liquid nitrogen at 77 K. After three cycles of freeze-pump-thaw operation, the tube was sealed with flame (the length of the tube was about 13.0 cm). The reaction mixture was heated at 120 °C for 3 days and the obtained orange powder was washed with DMF (15 mL×3), water (15 mL×3) and EtOH (15 mL×3) in turn. After removing the solvent under vacuum at 80 °C, TGDM was afforded as orange powder.

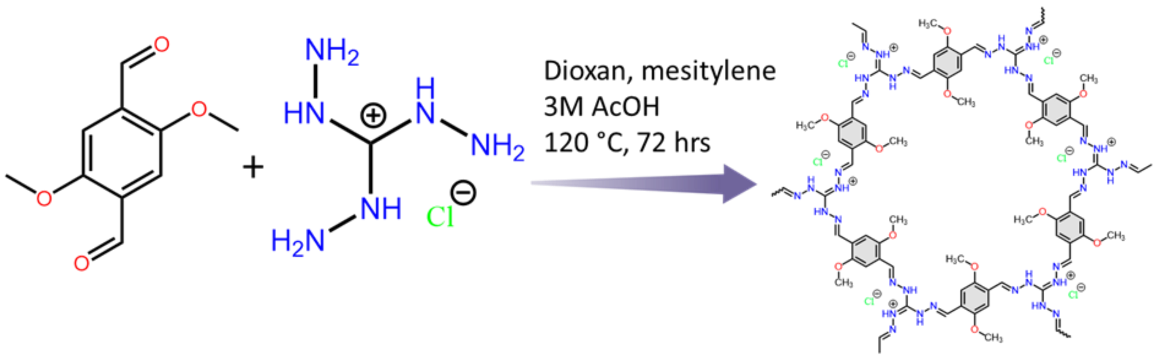

Scheme S3: Synthesis scheme of cationic covalent organic framework (iCOF).

S2.4 Synthesis of hybrid composite material (IPcomp-8): In a typical synthesis protocol, firstly a certain amount of NH_2 functionalized MOP was charged with TG_{Cl} (14.8 mg, 0.11 mM) and 2,5-dimethoxyterephthalaldehyde (29.1 mg, 0.15 mM) in a solution of deionized water/mesitylene/EtOH/3 M acetic acid (0.3/0.1/0.5/0.2 mL). The tube was flash frozen by liquid nitrogen at 77 K. After three cycles of freeze-pump-thaw operation, the tube was sealed with flame (the length of the tube was about 13.0 cm). The reaction mixture was heated at 120 °C for 3 days and the obtained dark orange powder was washed with DMF (15 mL×3), water (15 mL×3) and EtOH (15 mL×3). After that the compound was dipped in water and methanol for one day each, and finally was filtered off and dried.

Section S3: Oxoanion Capture Studies

S3.1 General Consideration: The aqueous solution of ReO_4^- was prepared by dissolving a certain amount of potassium salt of Re into deionized water and the different concentrations were obtained by diluting the stock solution with the proper amount of distilled water unless otherwise indicated. The pH values of the solutions were adjusted by HNO_3 and NaOH aqueous solution. The concentrations of ReO_4^- oxoanion during all the experiments were detected by ICP-AES and/or ICP-MS (for ultra-low concentrations) analysis. All the adsorption experiments were performed at ambient conditions. All the data has been collected three times through ICP analysis to plot the final adsorption results.

S3.2 TcO_4^- adsorption kinetics study: For the sorption study, 25 mg of IPcomp-8 was taken as solid phase, while 5 mL of aqueous phase containing TcO_4^- in specified pH (~5.4) made using nitric acid. The biphasic system was allowed to equilibrate for 3 hours followed by centrifugation for phase separation for a duration of 10 min. In case of pH variation experiments, the pH of the aqueous phase was varied from 2-8 and the temperature was kept constant 303 K. The aqueous phase before extraction and after extraction were collected and estimated (C_o and C_e) for Tc. The % removal values were estimated based on the equation below:

$D_t = (C_o-C_t)*100/C_o = (A_o-A_t)*100/A_o$, Where, D_t = removal efficiency (%), C_o = initial concentration, A_o = initial absorbance, C_t = final concentration, A_t = final absorbance at specific time.

The Tc-tetraphenyl arsonium chloride (TPAC) complex technique was used to determine the pertechnetate ion concentration in an aqueous solution. To determine the ^{99}Tc concentration in waste streams, ^{99}Tc radionuclide was analysed by precipitating with the TPAC complex and then measuring beta activity with a GM counter. Self-absorption of low energy beta particles through the precipitate introduces inaccuracy during beta activity counting. By employing its gross beta cut-off (95%) and an Al-absorber with a thickness of 100 mg cm^2, the concentration of ^{99}Tc was verified. To optimize the time of equilibration, the contact time between solid-liquid phase was varied from 5 min to 2 hours, while keeping the other experimental parameters same. The aqueous phase pH was kept pH ~5.4 for the experiments. In a similar fashion for understanding the sorption capacity, the experiments were performed at pH 5.4. The amount of TcO_4^- was varied in concentration range 25-250 mg L^{-1}. For all the experiments involving pH variation and capacity determination, the same solid to liquid ratio was kept and the time

of biphasic equilibration was kept as 2 hours, while 10 min was employed for centrifugation to achieve complete phase separation. The experiments were carried out at 300 K in a thermostated water bath with ±0.1 ^oC precision, procured from Lab Enterprise, Mumbai, Maharashtra. The centrifugation machine was also procured from Lab India.

S3.3 ReO_4^- adsorption kinetics studies: The standard procedure that we adopted for the adsorption kinetics experiments was as follows. The capture study of oxoanion ReO_4^- was performed in 15 mL glass vial equipped with magnetic stir bars with a constant stirring rate of 350 r.p.m. Firstly; 5 mg of material was taken in 5 mL of 25 ppm stock solution of oxoanion. After continuous stirring for different time at room temperature the solution was collected by a syringe with having 0.22 μm nylon membrane filter. The concentration of this filtrate was measured through ICP-AES and the kinetic adsorption study were measured at different time intervals. From the time-dependent study, we calculated the removal % and decreasing concentration of the oxoanion with time using the following equations:

Dt = (C_o-C_t) *100/C_o = (A_o-A_t) *100/A_o, Where, D_t = removal efficiency (%), C_o = initial concentration, A_o = initial absorbance, C_t = final concentration, A_t = final absorbance at specific time.

S3.4 ReO_4^- adsorption isotherm experiment: 5 mg of material was treated with 5 mL aqueous solution of ReO_4^- with rotation in ~15 mL glass vials having different concentrations of 25 ppm to 1000 ppm. After ~24 hours the filtrate was collected through syringe filter (0.22 μm) and measured the concentration through ICP-AES and further fitted the data with following equations,

Langmuir model, $Q_e = Q_m.C_e/(K_d+C_e)$; Where, C_e (ppm) and Q_e (mg g^{-1}) are the ReO_4^- concentration at equilibrium and amount of ReO_4^- adsorbed at equilibrium respectively. Q_m (mg g^{-1}) is the maximum amount of ReO_4^- per mass of adsorbent to form a complete monolayer. K_d (mg L^{-1}) is a constant to the affinity of the binding sites.

Freundlich model, $Q_e = K_f.C_e^{1/n}$; Where, K_f and 1/n are the Freundlich model constant, indicating capacity and intensity of adsorption respectively.

S3.5 Pseudo-second-order model: The pseudo-second-order equation can be expressed as follows: $t/q_t = 1/K_2q_e^2 + t/q_e$ Where, q_t and q_e represent the adsorbed amount (mg g^{-1}) at time and at equilibrium t (min), respectively, k_2 represent the Pseudo-second-order rate constant of

adsorption (g mg^{-1} min^{-1}). The experimental data was fitted using pseudo-second-order kinetic model.

S3.6 Distribution coefficient (K_d) value calculation: The distribution coefficient (K_d) value was used for the determination of the affinity and selectivity of sorbents for ReO_4^- oxoanion, is given by the equation:

$K_d = [V\{(C_o - C_e)/C_e\}]/m$; Where, V is the volume of the testing solution (mL), m is the amount of solid adsorbent (g), C_o is the initial concentration of ReO_4^-, and C_e is the equilibrium concentration of ReO_4^-.

S3.7 ReO_4^- adsorption capacity of IPcomp-8 after irradiation: The amount of ReO_4^- adsorbed per gram of material (irradiated by different doses of gamma ray) was determined by exposing 5 mg of material to 10 mL of ~1000 ppm concentrated solution of ReO_4^- for ~24 hours under stirring conditions at room temperature. After 24 hours, the composite material was filtered off and the filtrate was characterized through ICP-AES by diluting the solution. The adsorption capacity per gm was calculated using following equation:

$Q_t = (C_o - C_t) * V/m$;

Where, Q_t, C_o, C_t, V and m are the capacity of adsorbent, initial concentration of the ReO_4^- solution, final concentration of oxoanions at a specific time, volumes of the solution and mass of the material used for the adsorption study respectively.

S3.8 ReO_4^- capture study in presence of other competing ions: For testing the influence of competing ions, binary selectivity of salts such as, NaCl, NaBr, $NaNO_3$, $NaClO_4$, Na_2SO_4 and large excess of competing salt $NaNO_3$ and Na_2SO_4 (with varying concentration of 1, 10, 100, 1000 and 5000 ppm) were added to the ReO_4^- oxoanion aqueous solution and carried out the capture studies by following the aforementioned method. Conditions: $[Re]_{initial}$ = 25 ppm, contact time = 24 hours, and $m_{sorbent}/V_{solution}$ = 1 g/L. The capture studies were monitored through ICP-AES.

S3.9 pH-dependent TcO_4^- extraction study: The pH-dependent TcO_4^- extraction study was performed following the similar study as in the case of kinetics, at a certain time. The effect of pH on TcO_4^- removal performance was recorded in a wide range of pH (2 to 8) and compared with the data at pH 7 to check the relative performance. The variation of pH values was realized by the addition of either acid (HCl) or base (NaOH).

S3.10 Trace amount capture study: Trace amount (1 ppm) of ReO_4^- capture study was performed following the similar kinetic adsorption experiment described above. The kinetic adsorption study was measured at different particular time interval, taking ~1000 ppb stock solution of ReO_4^- in water. The ICP-MS experiments were carried out for analyse the removal efficiency of the materials.

S3.11 Recyclability test: The first cycle of ReO_4^- uptake in the recyclable experiment was performed according to the aforementioned adsorption protocol. After the first run of adsorption, the ReO_4^- containing material was regenerated by treatment with a saturated Na_2SO_4 aqueous solution. The resulting solid was dried and washed three times with pure deionized water. After being finally dried under vacuum, the resultant material was used for the next cycle adsorption experiments. Conditions: $[Re]_{initial}$ = 25 ppm, contact time = 24 hours, and $m_{sorbent}/V_{solution}$ = 1 g/L.

S3.12 Irradiation stability test and capture of 99mTc: The materials were subjected to the gamma irradiation using GB5000, FTD, BRAC. Depending on the dose rate and required dose, the materials were placed inside the gamma chamber for predefined time. 60Co source has been employed in the present case for achieving the gamma dose. Subsequently, with gamma irradiated materials, the uptake studies were performed. 99mTc has been chosen as the radiotracer for the experiments and the determination was done by gamma spectrometry. 10 mg of compounds were allowed to equilibrate with 1.5 mL of the aqueous phase containing Tc radiotracer at different feed acidity. Subsequently, it was allowed to equilibrate in a thermostated water bath at ambient temperature (27±0.1°C) for 3 hours for biphasic mass transfer. The 5 min centrifugation was performed for complete phase separation. Then, suitable aliquots of the aqueous phase was analyzed by gamma spectrometry for Tc determination. From the initial counts before extraction the uptake values were calculated.

S3.13 ICP Calibration: The calibration curve was recorded before each ICP measurement using various standard samples prepared by dilution of ReO_4^- oxoanion salt aqueous solution of a known concentration with 2% w/w HNO_3. The calibration curves indicated the regression line with high correlation coefficient (R^2 = 0.999, R^2 = 0.998). All the ICP data were conducted triplicate. Whereas, few critical ICP data (such as kinetics and capacities) has been collected and verified from two different instruments.

Section S4: Structural Characterization of Synthesized Materials

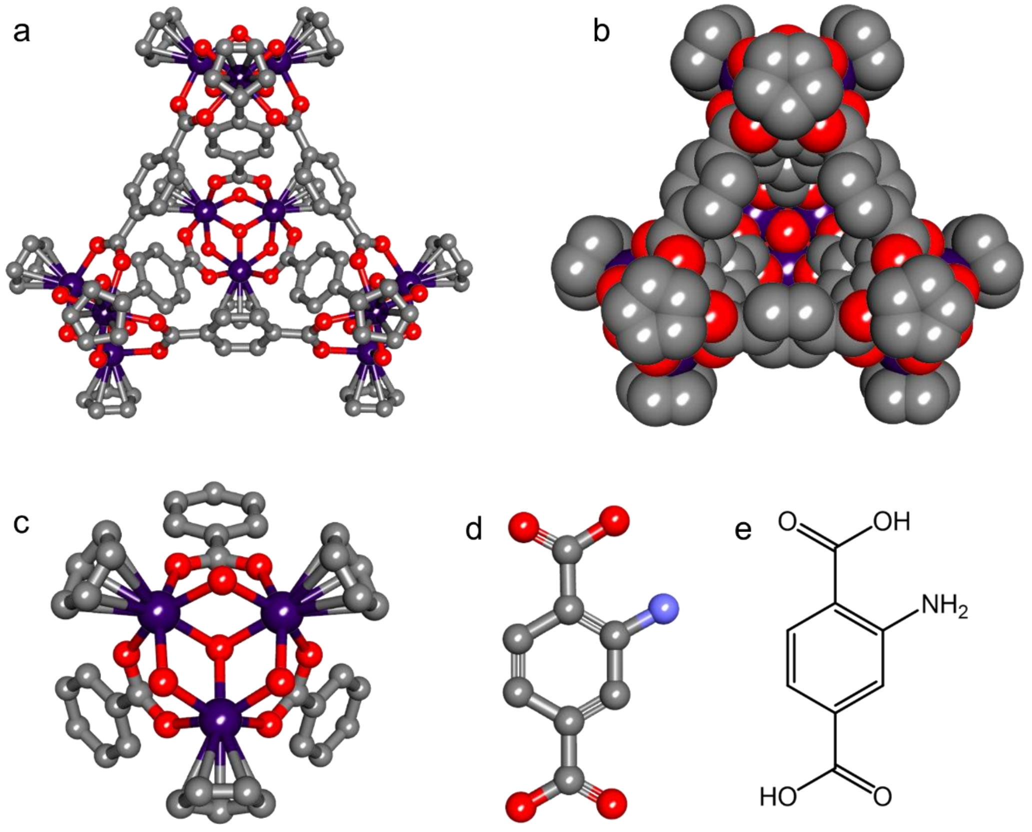

Fig. S1: (a, b) Structure of amino functionalized metal-organic polyhedra, Zr(IV)-MOP with its structural features of (c) Zr(IV) metal-based secondary building unit (SBU) and (d, e) 2-aminoterepthalic acid ligand. (Color code: gray: carbon, red: oxygen, light blue: nitrogen, dark violet: zirconium, hydrogen is omitted for clarity).

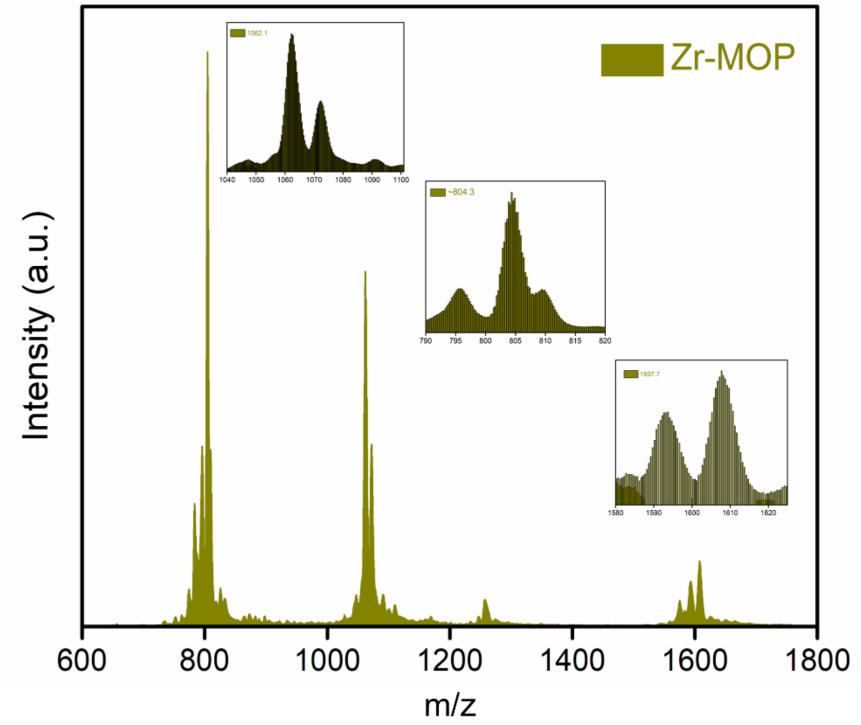

Fig. S2: ESI-TOF-MS analysis of as-synthesized NH_2-MOP.

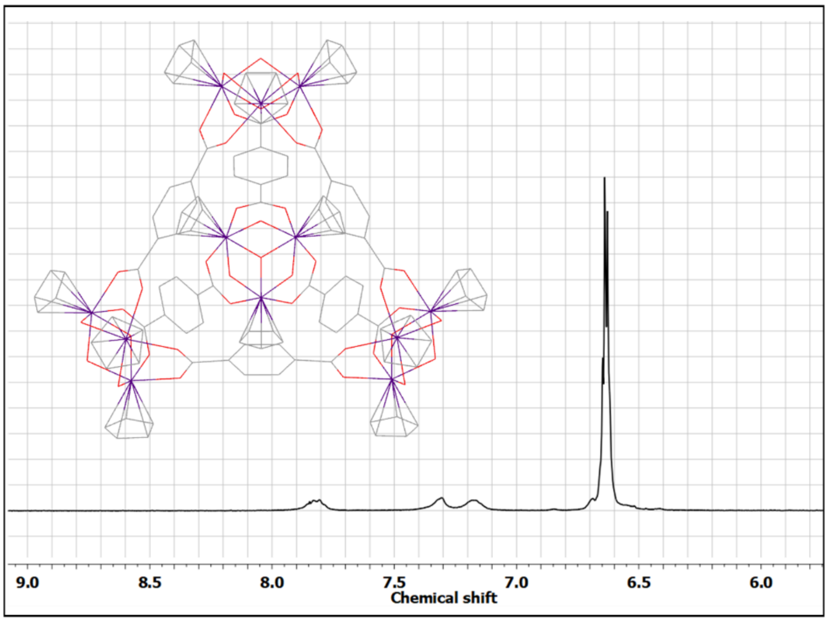

Fig. S3: ^{1}H NMR spectra of the NH_2-MOPs in MeOD and D_2O.

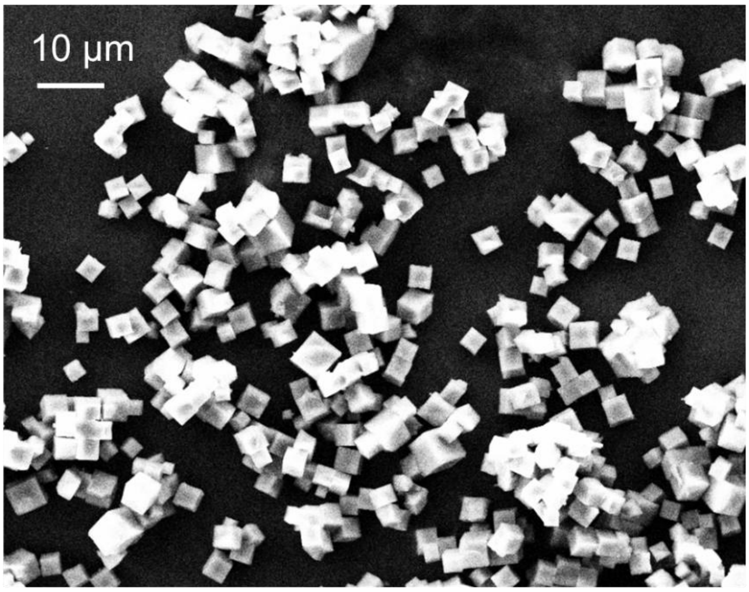

Fig. S4: FESEM image of the MOPs, indicated highly cubic microcrystalline morphology.

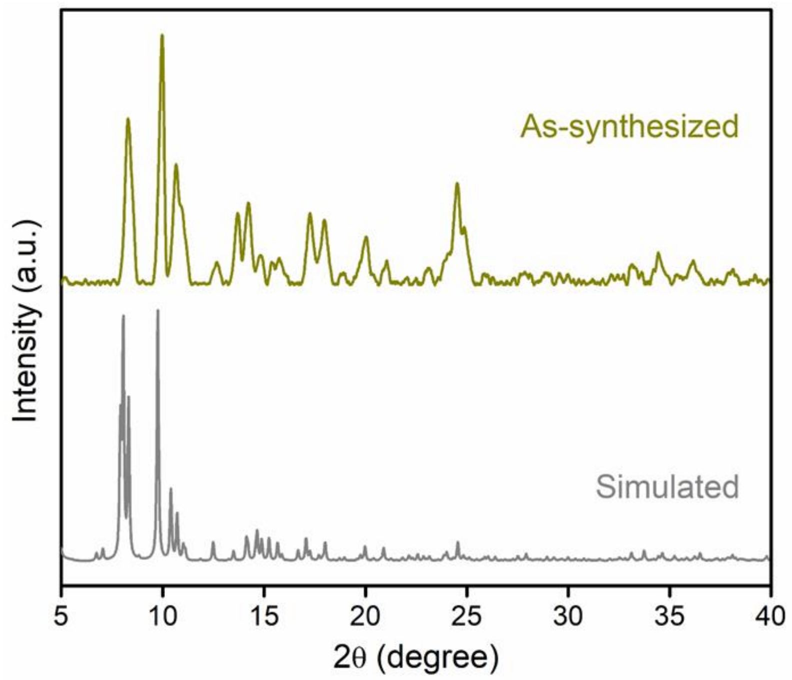

Fig. S5: PXRD analysis of the NH_2-MOP indicated formation of bulk phase purity in as-synthesized phase.

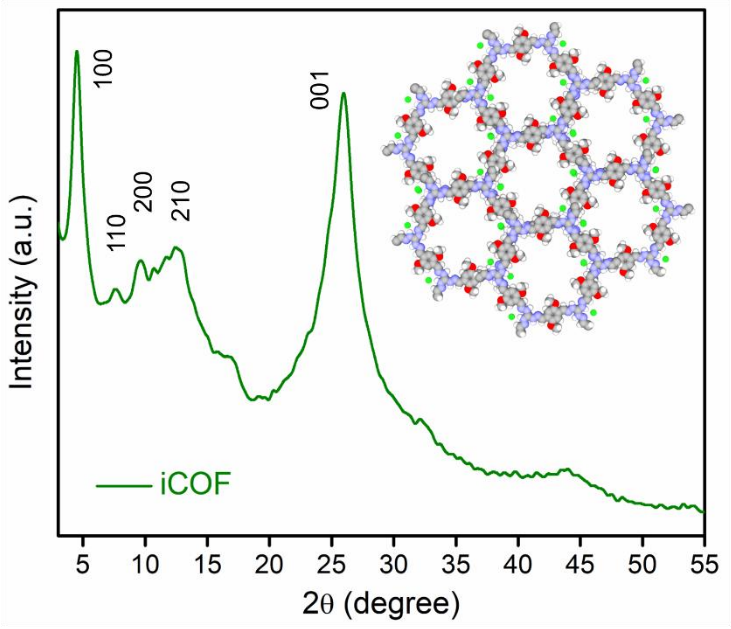

Fig. S6: PXRD spectra of the iCOF.

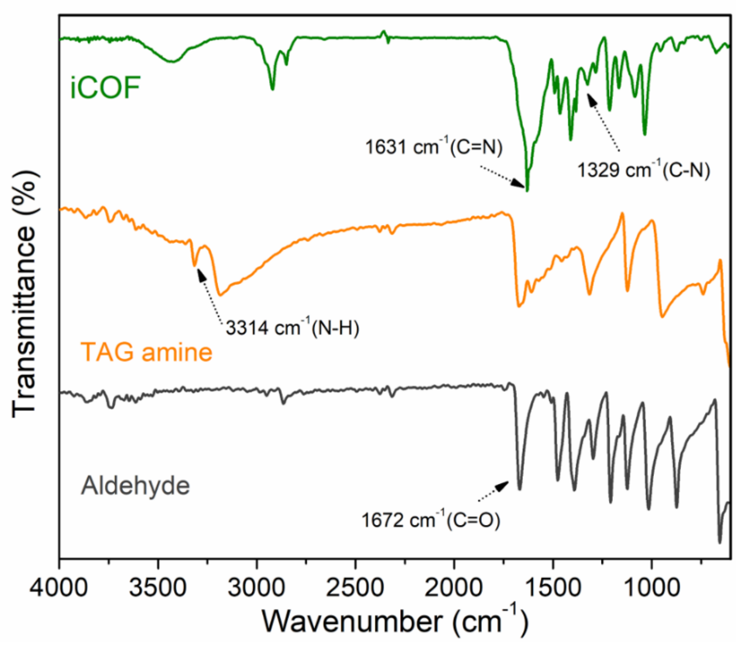

Fig. S7: FTIR spectra of the iCOF with its precursors.

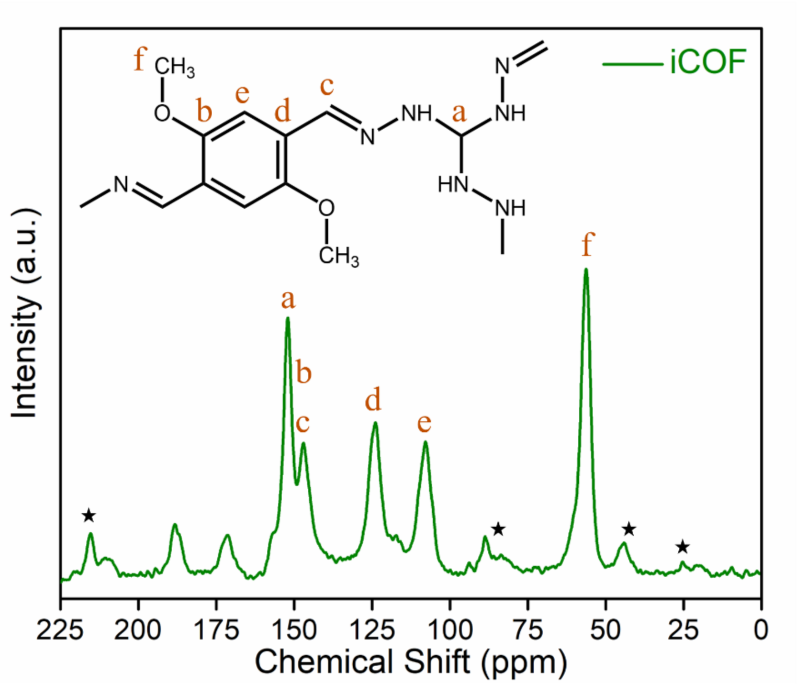

Fig. S8: Solid-state ^{13}C CP-MAS NMR spectra of the iCOF. Asterisks denote spinning sidebands.

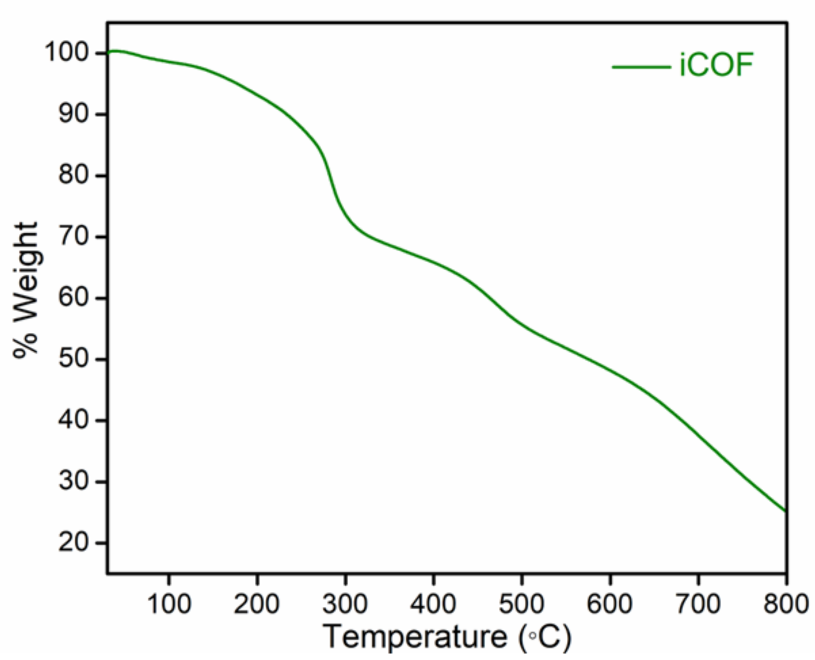

Fig. S9: TGA profile of the iCOF.

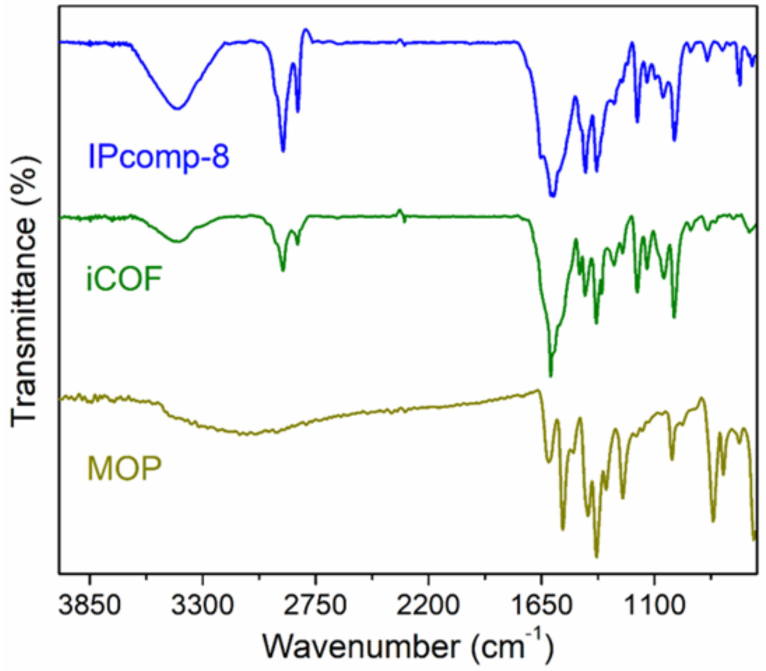

Fig. S10: FTIR spectra of IPcomp-8 along with pristine MOP and iCOF.

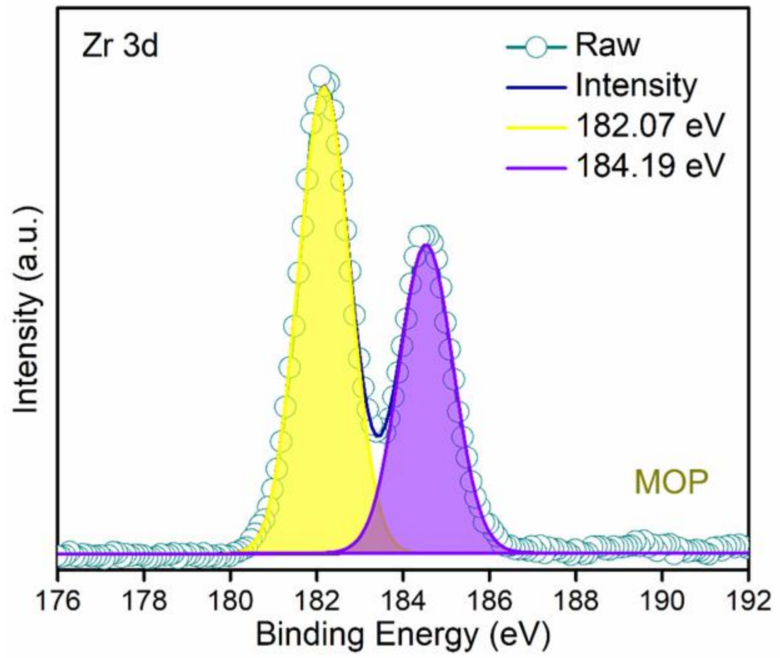

Fig. S11: Deconvoluted XPS spectra of Zr 3d orbital of pristine MOP.

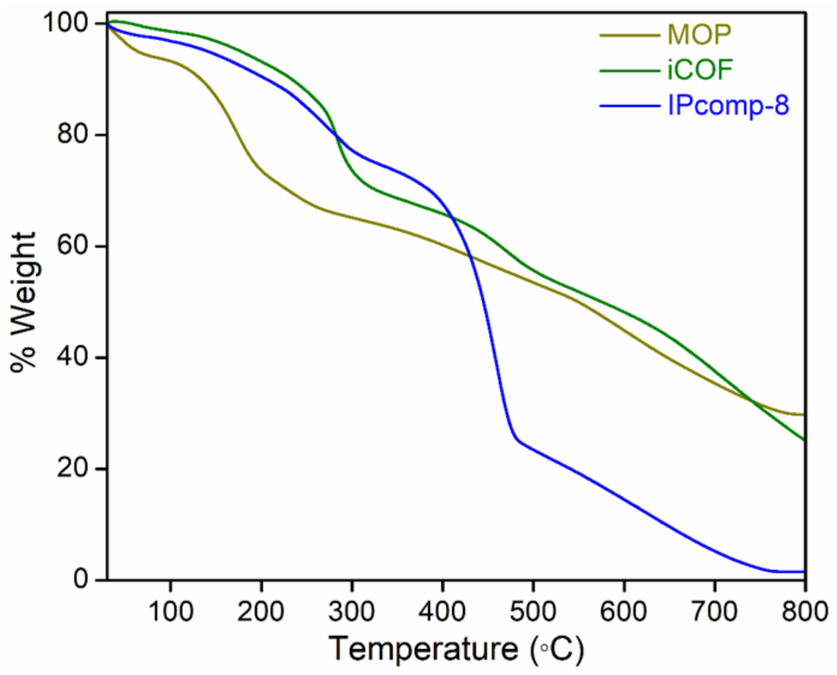

Fig. S12: TGA profile of the MOP, iCOF and IPcomp-8 composite.

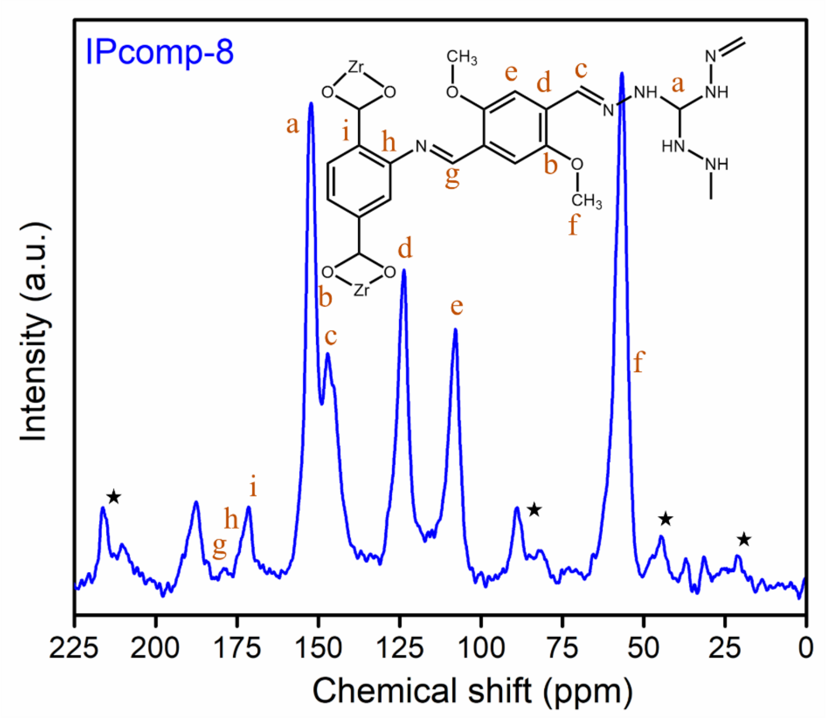

Fig. S13: ^{13}C solid-state CP-MAS NMR spectrum of IPcomp-8. Asterisks denote spinning sidebands.

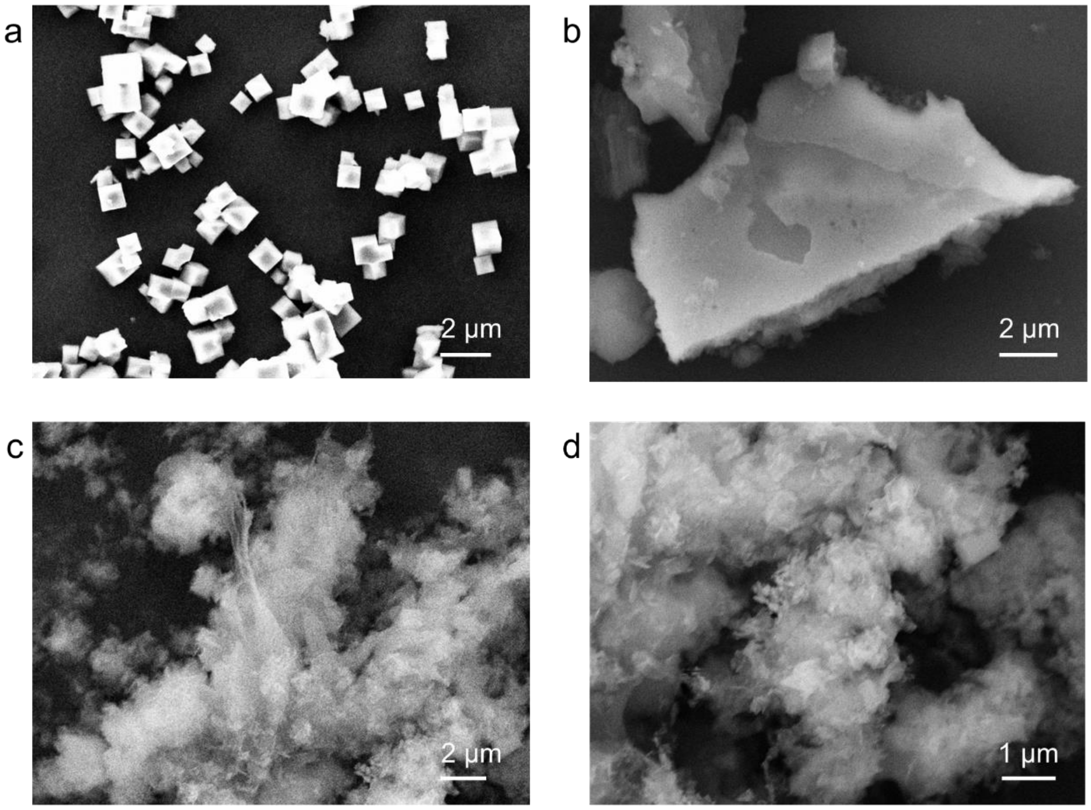

Fig. S14: FESEM images of (a) MOP; (b) iCOF and (c, d) IPcomp-8 composite.

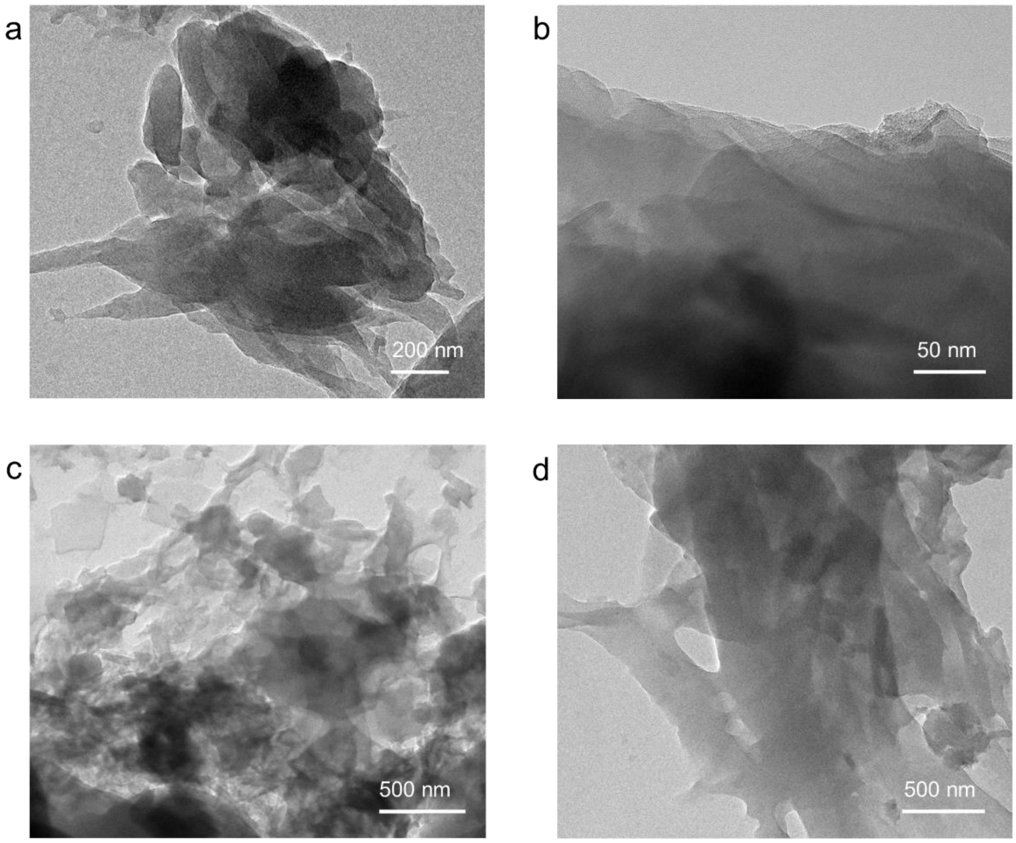

Fig. S15: TEM images of (a, b) iCOF and (c, d) IPcomp-8 composite.

Table S1: EDX data from FESEM experiment of Zr-MOP.

Element	Weight%	Atomic%
C K	46.66	67.19
N K	3.88	4.79
O K	19.86	21.44
Cl K	3.64	1.67
Zr L	25.95	4.91
Total	100	100

Table S2: EDX data from FESEM experiment of iCOF.

Element	Weight%	Atomic%
C K	50.98	57.68
N K	26.41	25.62
O K	17.23	14.64
Cl K	5.38	2.06
Total	100	100

Table S3: EDX data from FESEM experiment of IPcomp-8 (area-1).

Element	Weight%	Atomic%
C K	41.79	51.35
N K	21.05	21.61
O K	30.09	24.31
Cl K	1.99	0.67
Zr L	5.08	2.06
Total	100	100

Table S4: EDX data from FESEM experiment of IPcomp-8 (area-2).

Element	Weight%	Atomic%
C K	43.06	55.90
N K	20.14	17.88
O K	25.44	22.13
Cl K	2.78	0.98
Zr L	8.58	3.11
Total	100	100

Table S5: EDX data from FESEM experiment of IPcomp-8 (area-3).

Element	Weight%	Atomic%
C K	40.12	56.61
N K	19.98	16.21
O K	25.71	21.22
Cl K	4.01	1.47
Zr L	10.18	4.49
Total	100	100

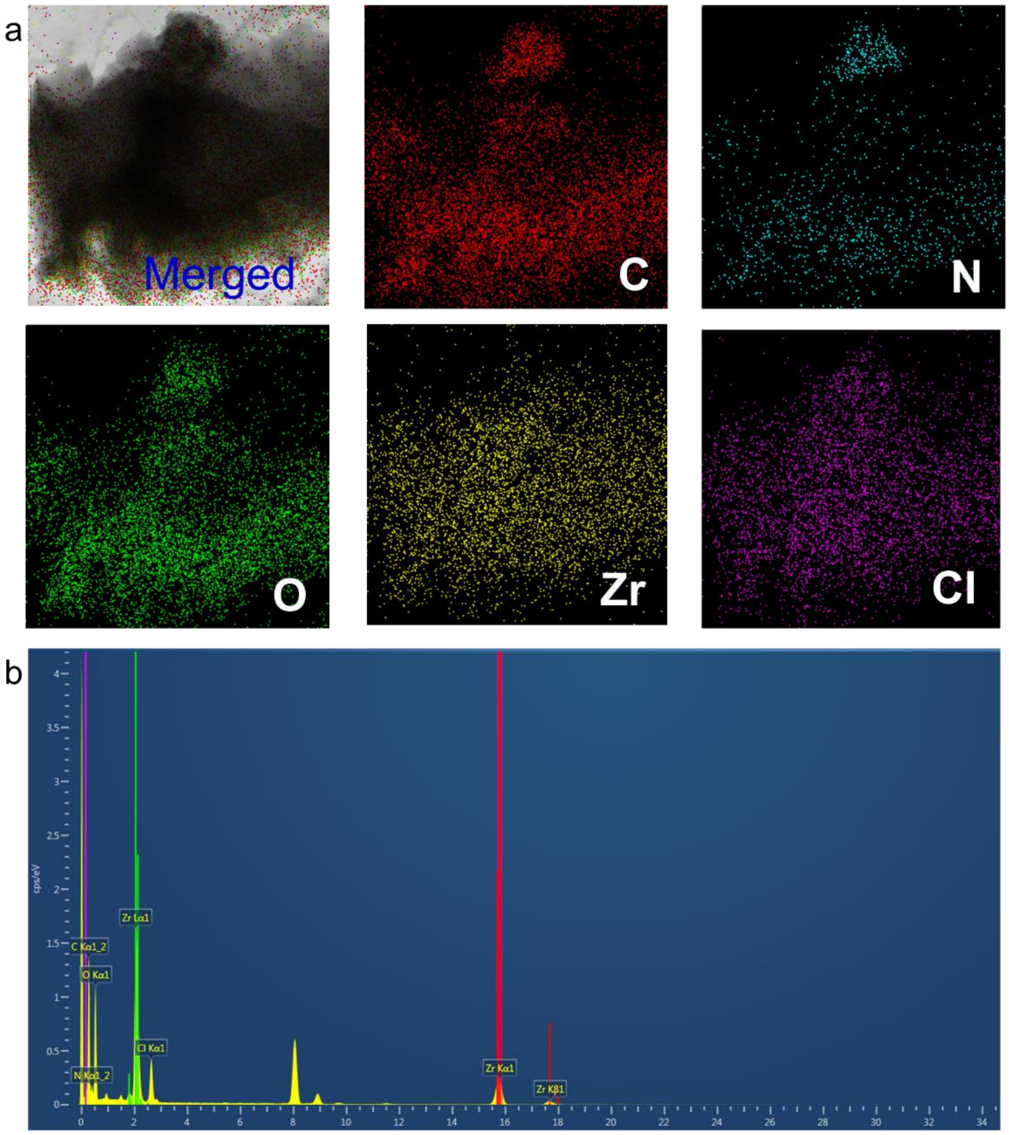

Fig. S16: (a) Elemental mapping image and (b) EDX profile of IPcomp-8.

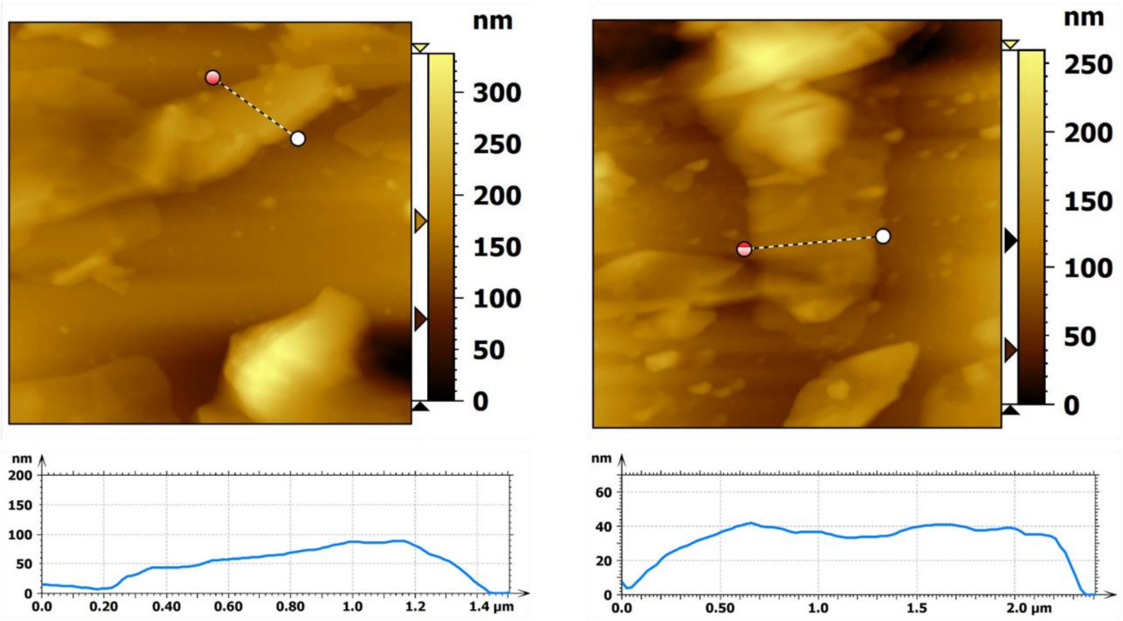

Fig. S17: AFM images and height profiles of pristine iCOF.

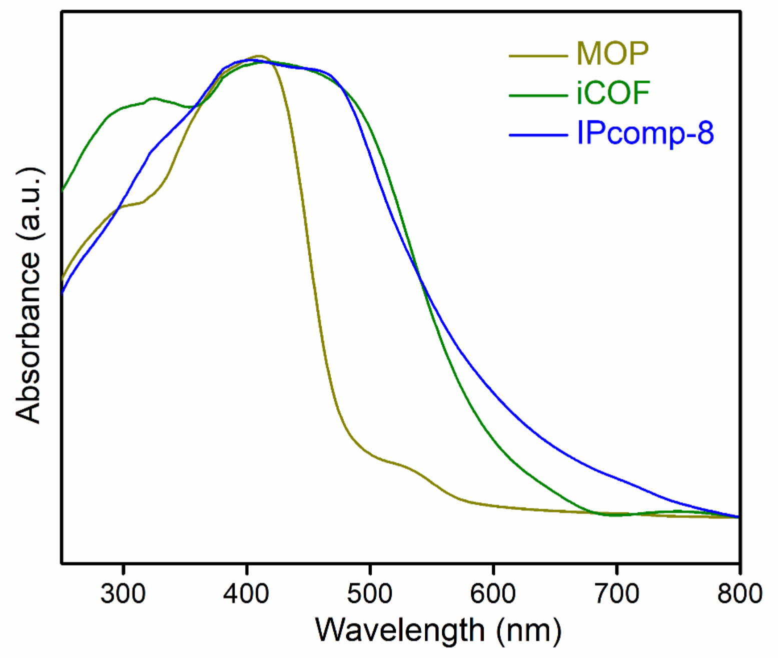

Fig. S18: UV-vis diffuse reflectance spectroscopy (DRS) spectra of the MOP, iCOF and IPcomp-8.

Section S5: Stability and Leaching Test

Supporting note 1: To investigate the stable existence of the Zr-MOP in the synthetic condition of the composite as well as to test the leaching possibility of the Zr-MOP from the composite before and after the metal oxoanions capture test, we carried out the following series of control experiments. For this, we applied nuclear magnetic resonance (NMR) spectroscopy, high-resolution mass spectrometry (HRMS) and UV-vis experiments. At first, the NMR, HRMS and UV-vis spectra of as-prepared hybrid composite, without washing with any solvent was recorded. The ^{1}H NMR, ESI-TOF-MS and UV-vis spectra of the supernatant (collected by treating the composite with aqueous methanolic solution) (in case of ^{1}H NMR, treating with methanol d^4 and deuterium oxide (D_2O)), indicated the presence as well as stable existence of the MOP molecules within the synthetic condition of the composite (Fig. S19). As revealed by the ESI-TOF-MS and UV-vis spectra of the supernatant, the MOP molecules exhibited similar characteristic with the pristine MOPs (Fig. S19). Such appearance of peaks related to the MOP molecules in these spectra can be explained by the easy release of the non-covalently bounded excess MOPs present on the surface of the composite. However, after repeated thorough washing with aqueous methanolic solution of the composite, all these spectra were again recorded. The ^{1}H NMR, ESI-TOF-MS and UV-vis spectra of the washed composite treated supernatant (same as previous) exhibited no presence of MOPs, which ruled out the possibility of existence of any excess non-covalently bounded MOPs on the surface of composite as well as no liberation of MOPs from the iCOF of the hybrid (IPcomp-8) (Fig. S20). Interestingly, when the composite was treated/digested with NaOH + D_2O, the appearance of relevant peaks of the corresponding organic linker of the MOP (i.e. 2-amino terephthalate) in the ^{1}H NMR spectra support the presence of covalently joined amino functionalized cationic MOP in the composite material (Fig. S21). All these results indicated the stability and existence of the MOP molecules in the hybrid composite and thus successful formation of IPcomp-8. In addition to this, the ICP-OES data of the digested sample of IPcomp-8 found to exhibit a loading of total ~3.47 wt% of MOP in the composite material.

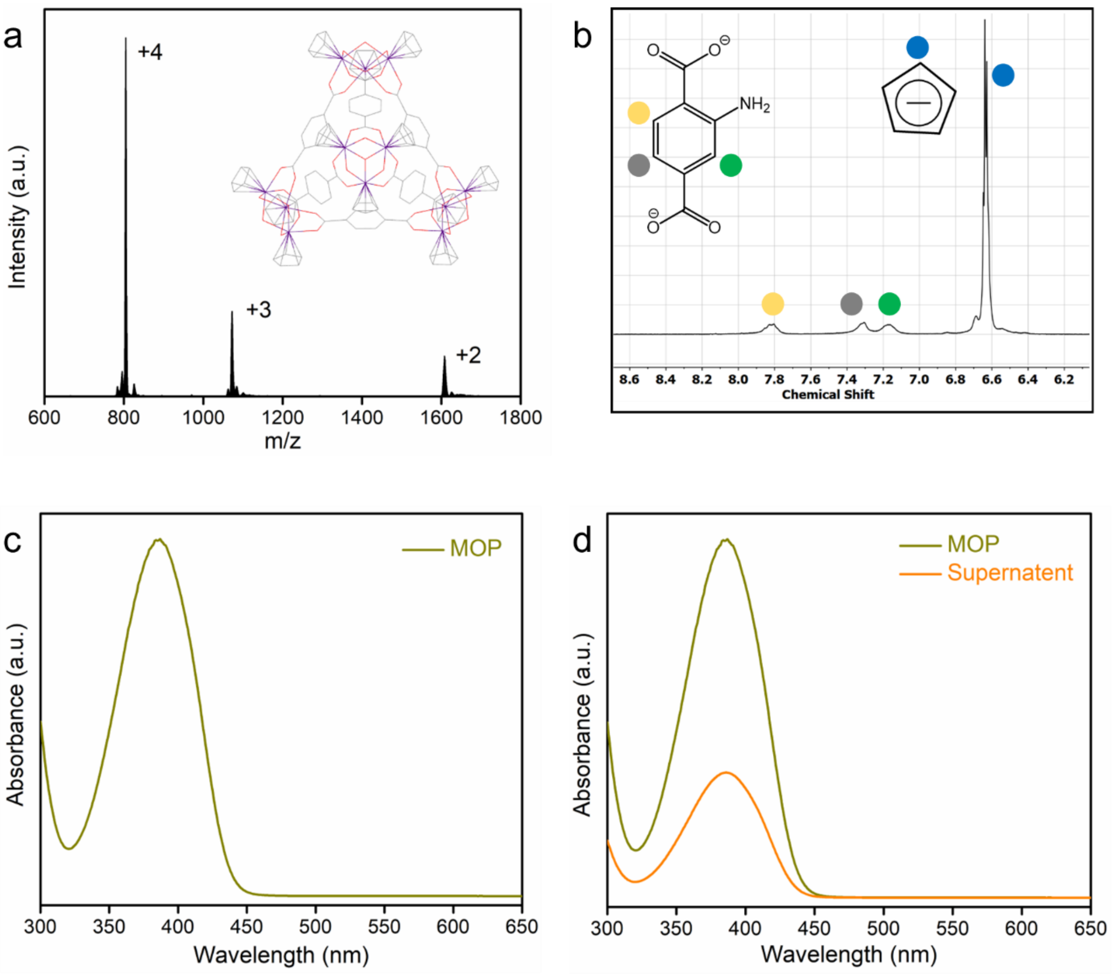

Fig. S19: (a) ESI-TOF-Mass spectra of NH_2-MOP (in aqueous methanol), (b) ^1H NMR analysis of NH_2-MOP (in MeOD + D_2O), (c, d) UV-vis spectra of the pristine NH_2-MOP and NH_2-MOP present in the supernatant, collected by treated IPcomp-8 with aqueous methanolic solution, respectively. All these spectra indicated the presence of NH_2-MOP in the solution.

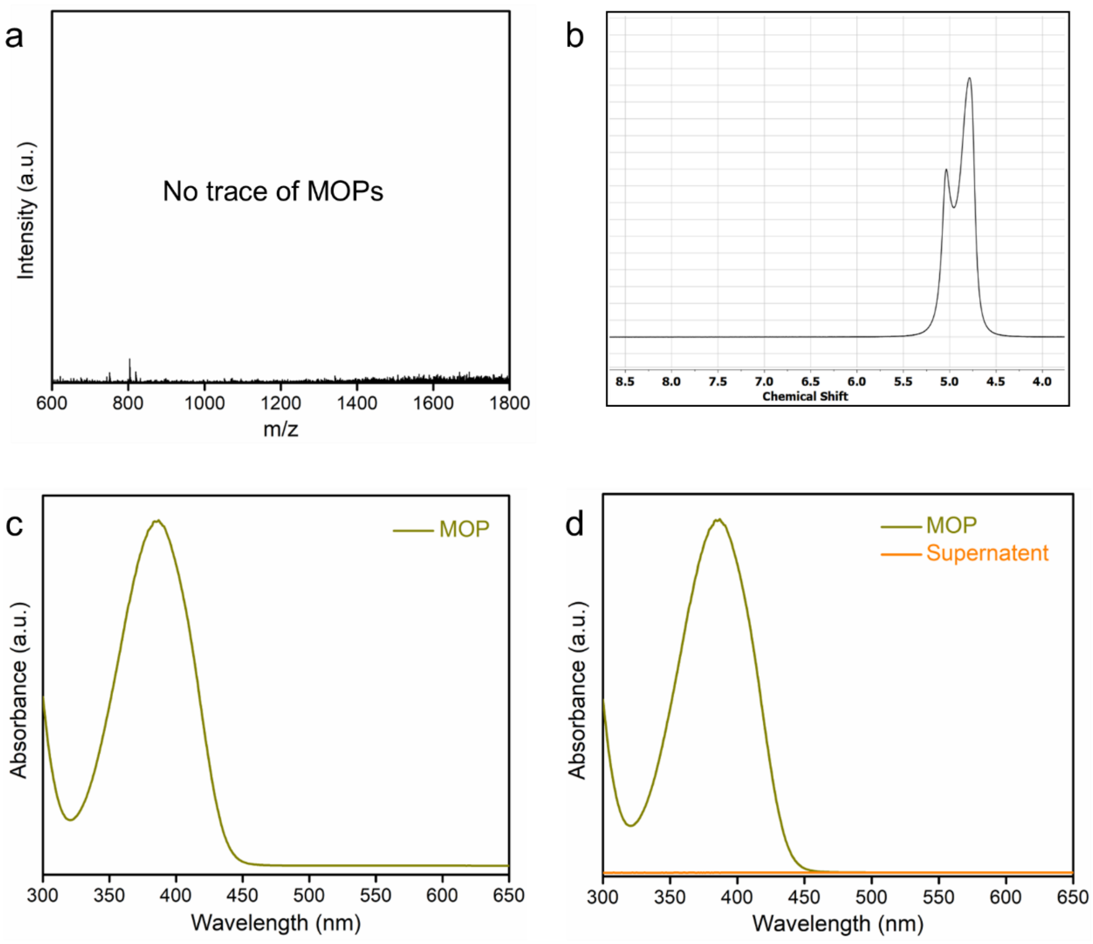

Fig. S20: (a) ESI-TOF-Mass spectra (in aqueous methanol), (b) ^{1}H NMR analysis (in MeOD + D_2O), (c, d) UV-vis spectra of the pristine NH_2-MOP and the supernatant, collected by treated IPcomp-8 with aqueous methanolic solution, respectively. These spectra were recorded after thorough washing the composite with aqueous methanolic solution. All these spectra indicated no presence of NH_2-MOP in the solution.

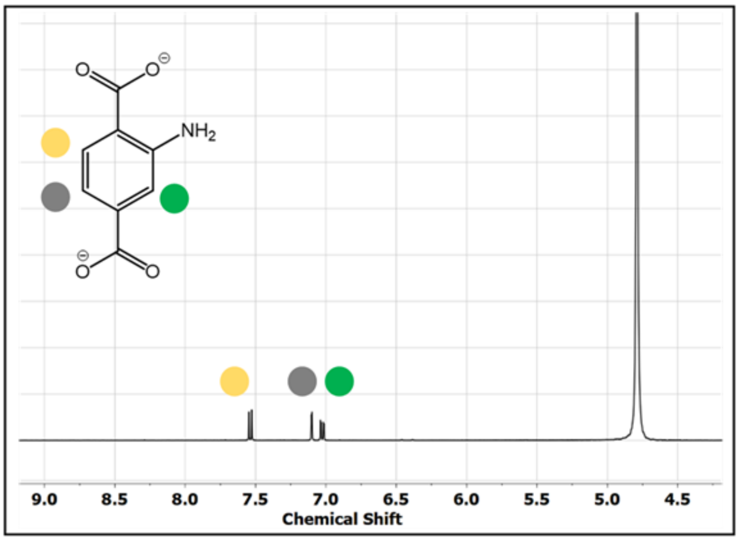

Fig. S21: ^{1}H NMR analysis of digested IPcomp-8 (in NaOH/D_2O), The spectra was recorded after digested the composite. This data indicated the presence of organic linker of the NH_2-MOP in the digested sample of the composite.

In order to further confirm the covalent bonding between the amino (-NH_2) functionalized MOP and triaminoguanidine moiety based ionic COF (iCOF) structure, a model compound has been synthesized by reacting the NH_2-MOP with 4-anisaldehyde the similar reaction condition to that of hybrid composite (IPcomp-8) synthesis. The following ^{1}H NMR data displayed the reaction of NH_2-MOP with aldehyde as indicated by the appearance of new peaks in the chemical shift range of ~7.4-7.7 ppm (Fig. S22). Such observation was found to match with the previously reported literature.[3]

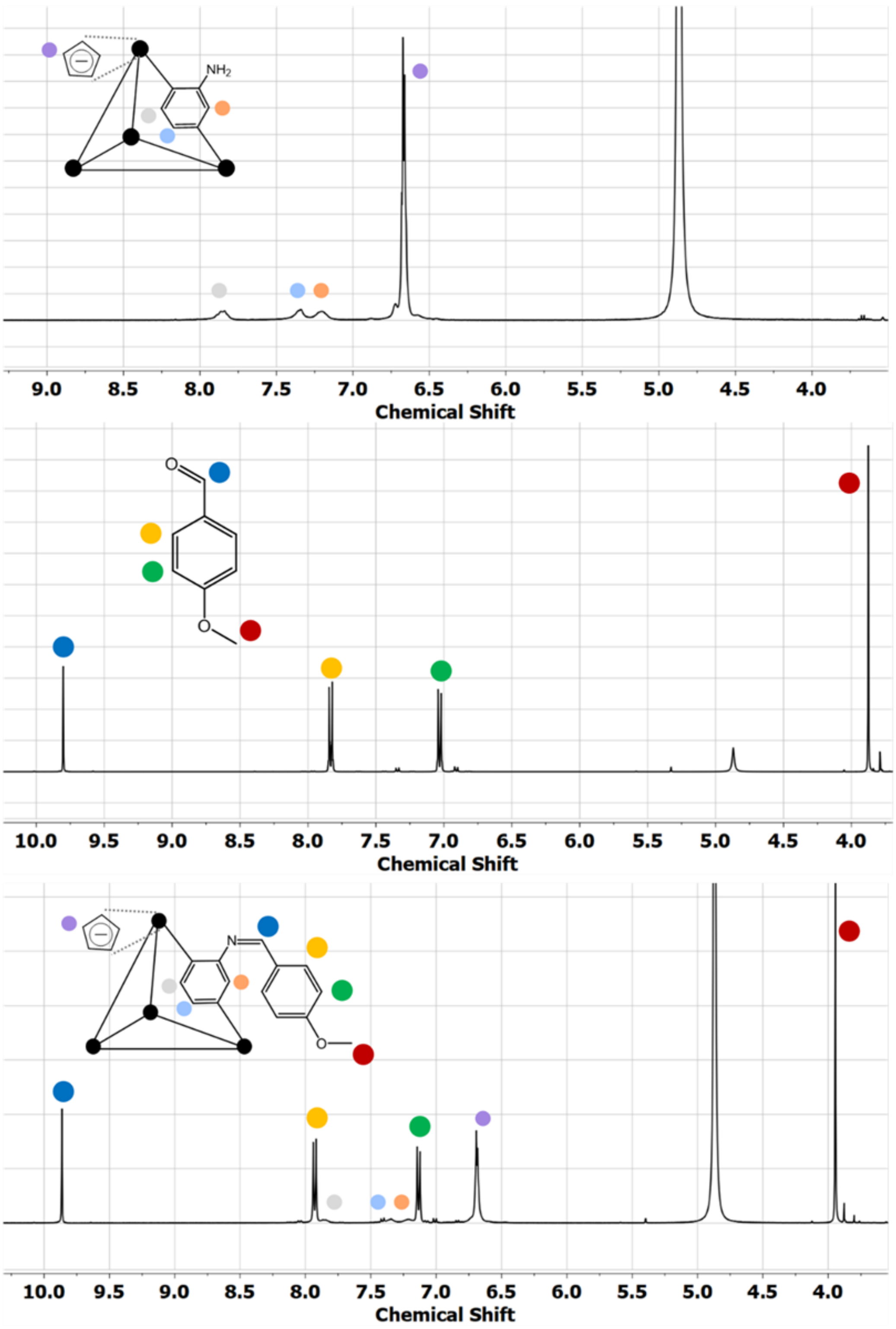

Fig. S22: ^{1}H NMR analysis of pristine NH_2-MOP, 4-anisaldehyde, and the model compound synthesized by reacting 4-anisaldehyde with the NH_2-MOP.

Supporting note 2: To further investigate the stable existence of the Zr-MOP within the iCOF matrix, the chemical composition and elemental state were analysed by X-ray photoelectron spectroscopy (XPS). The full XPS survey spectra indicate the presence of carbon, oxygen, nitrogen, chlorine, and zirconium in the composite (IPcomp-8) (Fig. S23). The high-resolution XPS spectrum of the deconvoluted Zr 3d of all the relevant compounds are shown in Fig. S24. For IPcomp-8, two major peaks located at 182.38 eV and 184.68 eV, are assigned to the Zr $3d_{5/2}$ and Zr $3d_{3/2}$, core energy level, respectively. On the other hand, in case of MOP, these two peaks are located at 182.07 eV and 184.19 eV, respectively. Notably, these near similar electronic state with close binding energies of Zr(IV) in the MOP with that of IPcomp-8 demonstrates that the zirconium signals in the composite (IPcomp-8) come indeed from Zr of the MOP structure. However, a slight shifting in the binding energies of Zr 3d of these two materials indicates that there is an interaction between Zr(IV) of the MOP with iCOF matrix. Interestingly, when compared with Zr 3d XPS spectra of $ZrCp_2Cl_2$ metal salt with Zr 3d spectra of both MOP and IPcomp-8, a significant shift in the binding energies was clearly observed (Fig. S24). This further evidences the coordination of Zr-O interaction in the MOP as well as IPcomp-8. All these results suggest the stable existence of the Zr-MOP within the iCOF matrix of IPcomp-8.

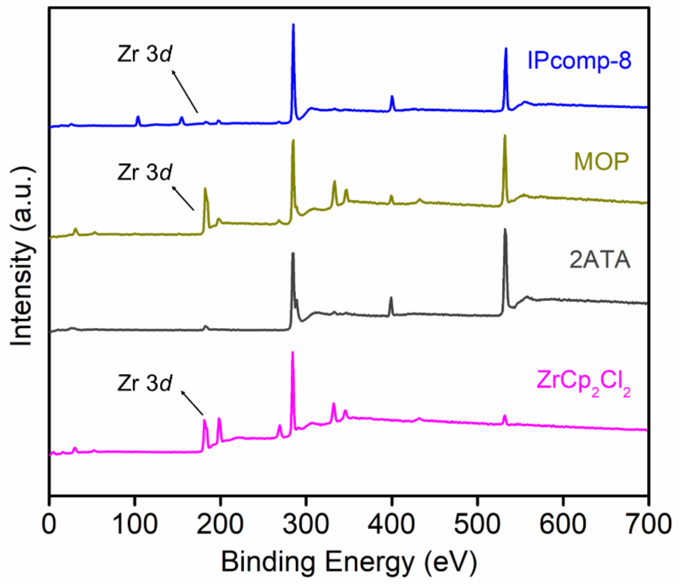

Fig. S23: XPS survey spectra of $ZrCp_2Cl_2$, 2-amino-terepthalic acid (2ATA), pristine Zr-MOP, and IPcomp-8 composite.

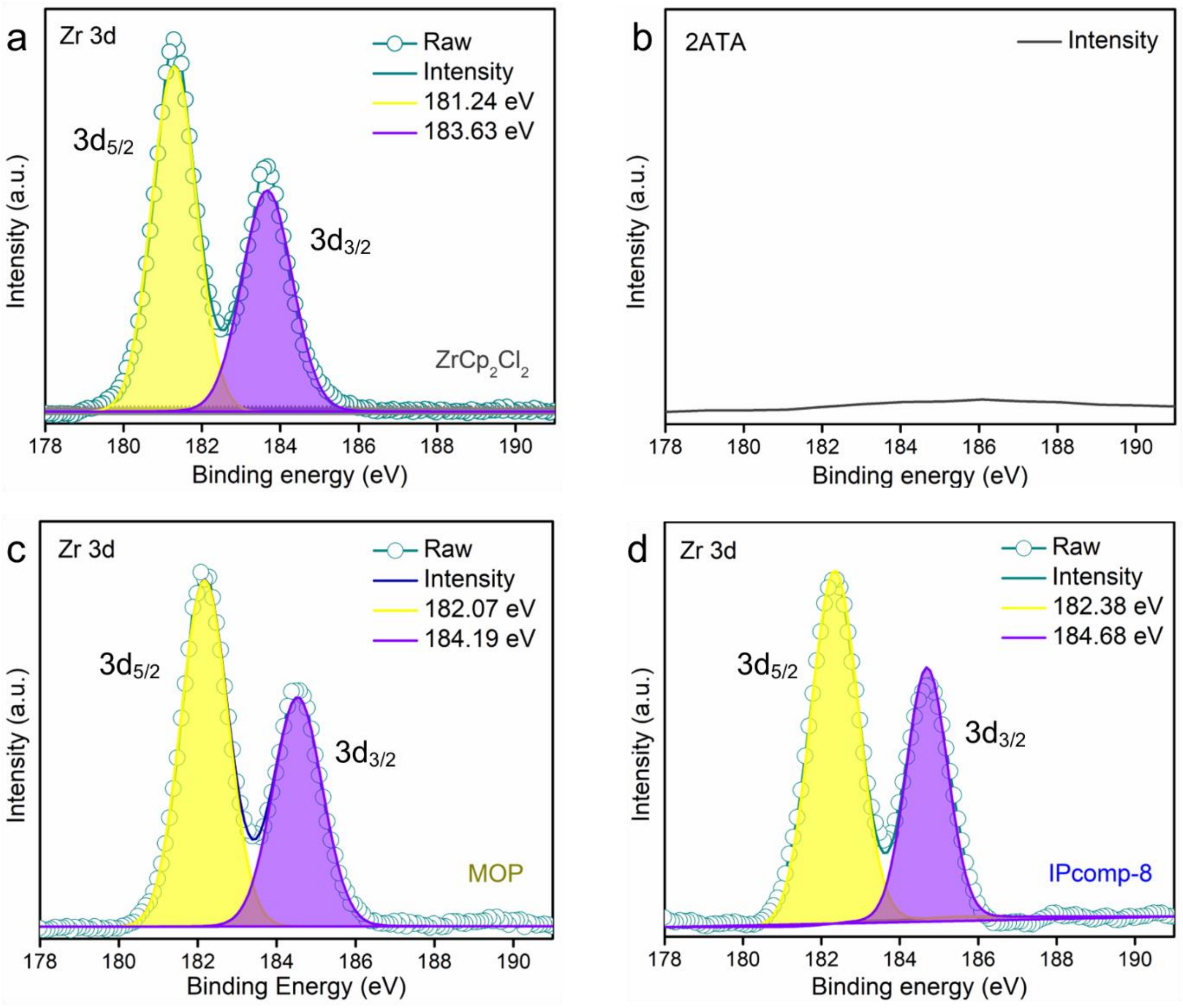

Fig. S24: High-resolution XPS spectra of deconvoluted Zr 3d in different samples; (a) $ZrCp_2Cl_2$, (b) 2-amino-terepthalic acid, (c) pristine Zr-MOP, and (d) IPcomp-8 composite.

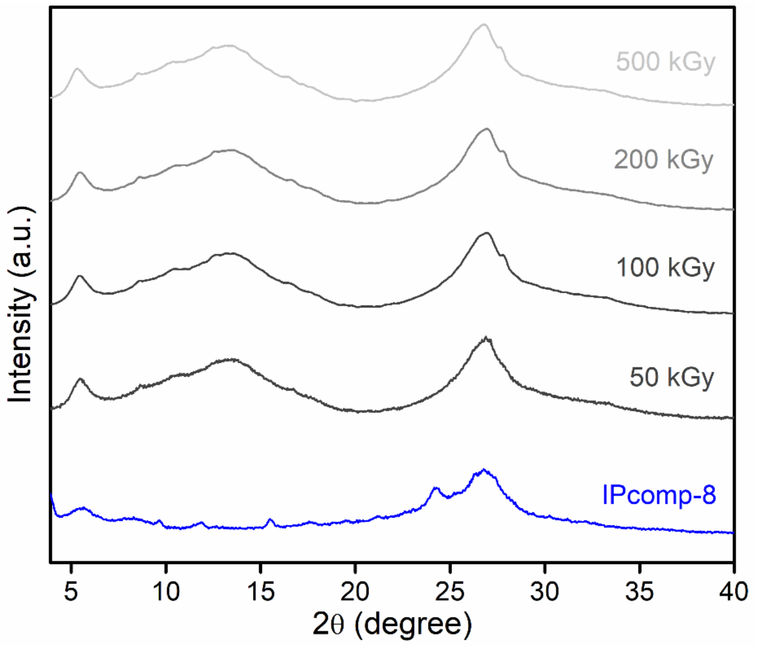

Fig. S25: PXRD profile of IPcomp-8 before and after irradiation of different doses of gamma radiation.

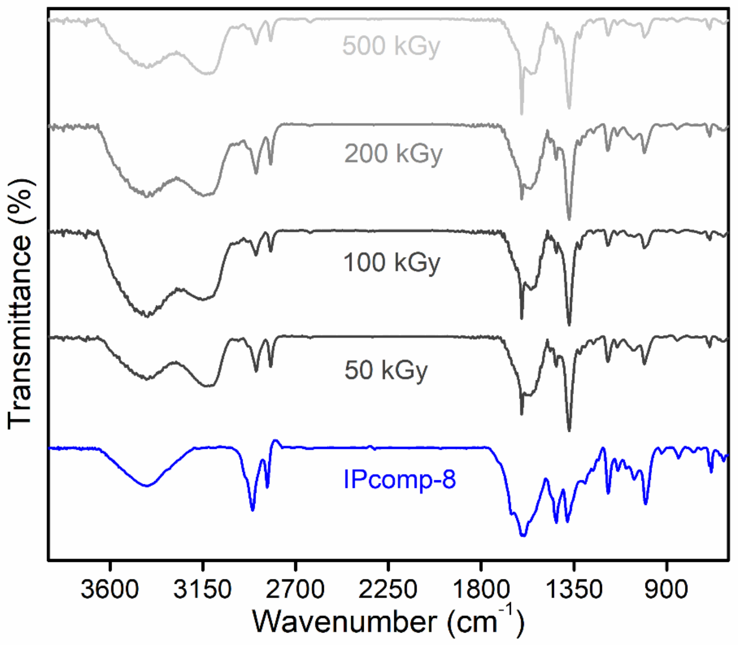

Fig. S26: FT-IR spectra of IPcomp-8 before and after irradiation of different doses of gamma radiation.

Section S6: Oxoanion Capture Studies

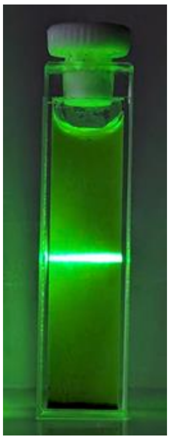

Fig. S27: Digital image of Tyndall effect exhibited by IPcomp-8 in aqueous medium.

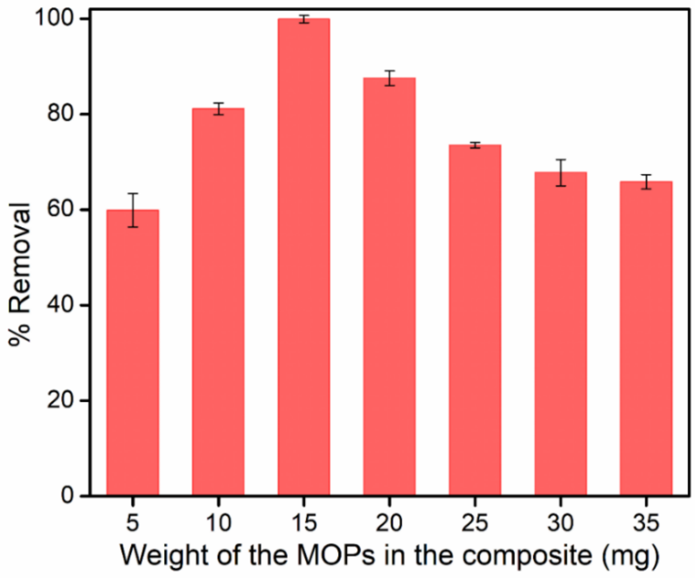

Fig. S28: Optimization result of ReO_4^- capture study with various composite synthesized with different amount of MOPs. Condition: ~25 ppm stock ReO_4^- solution, treatment time 5 min, sorbent weight = 2 mg, volume = 2 mL.

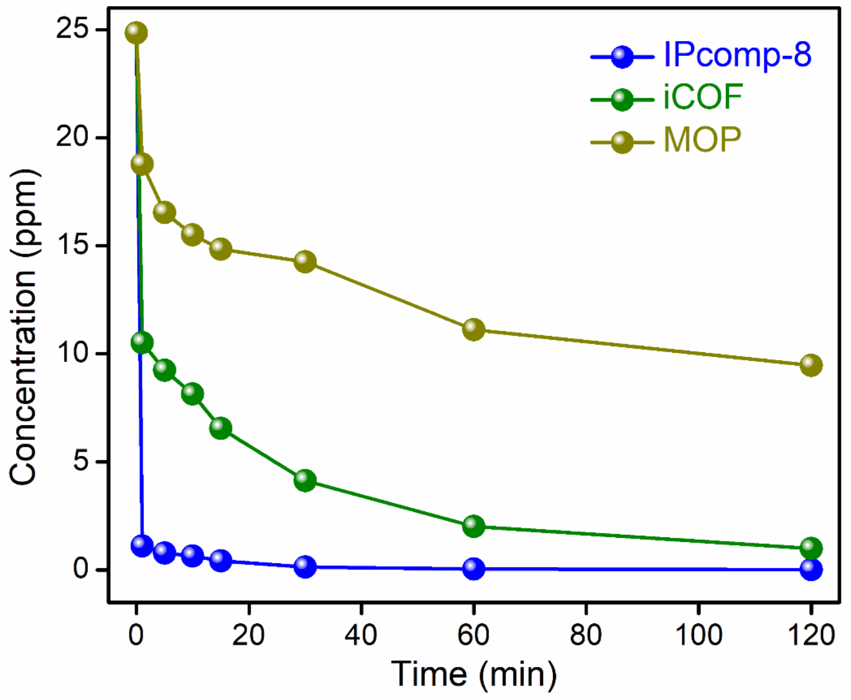

Fig. S29: Decrease in the concentration of ReO_4^- anion in different contact times by IPcomp-8, iCOF and MOP.

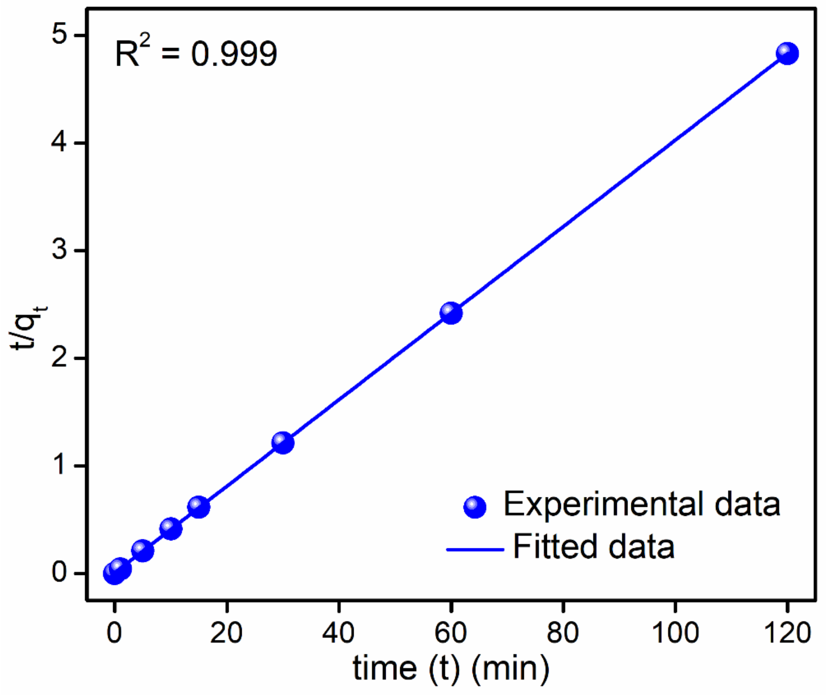

Fig. S30: Pseudo-second order model fitting curve of ReO_4^- aqueous solution by IPcomp-8 (rate constant K = 0.2739 g.mg^{-1}.min^{-1}).

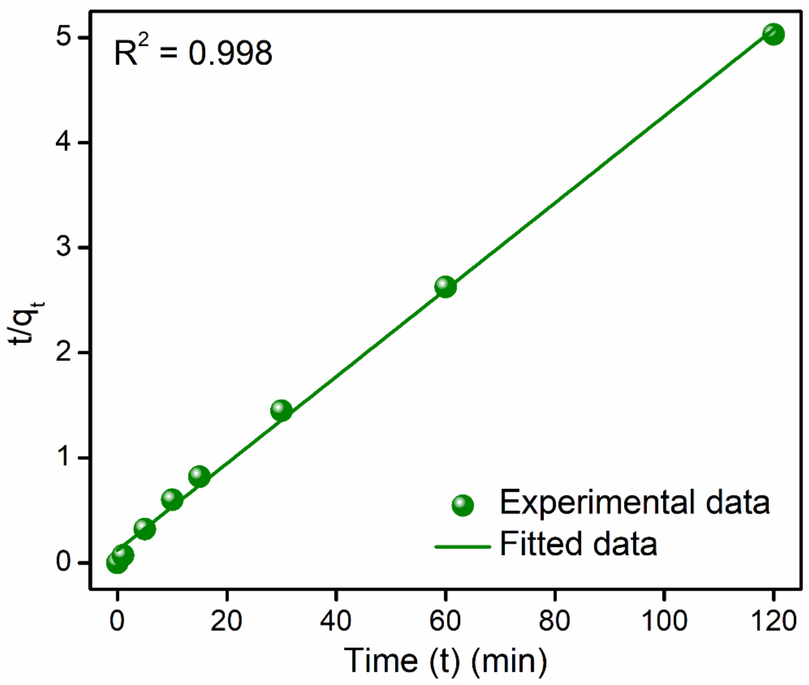

Fig. S31: Pseudo-second order model fitting curve of ReO_4^- aqueous solution by iCOF (rate constant K = 0.0144 g.mg^{-1}.min^{-1}).

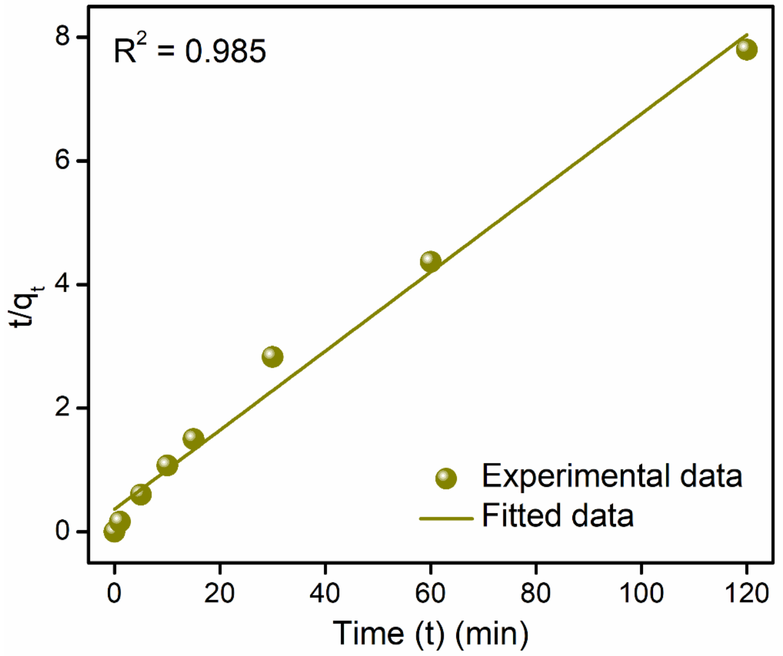

Fig. S32: Pseudo-second order model fitting curve of ReO_4^- aqueous solution by MOP (rate constant K = 0.0109 g.mg^{-1}.min^{-1}).

Table S6: ReO_4^- adsorption capacity of IPcomp-8 before and after irradiation of different doses of gamma ray.

Irradiation	ReO_4^- capacity (mg/g)
0 kGy	632
100 kGy	553
200 kGy	509

Table S7: K_d values of IPcomp-8 for ReO_4^- in the presence of other competing anions.

Anions	K_d values
ReO_4^-	5.71 x 10^6
ReO_4^- + Cl^-	0.63 x 10^6
ReO_4^- + NO_3^-	3.72 x 10^6
ReO_4^- + CO_3^{2-}	1.15 x 10^7
ReO_4^- + Br^-	1.02 x 10^6
ReO_4^- + SO_4^{2-}	1.17 x 10^7
ReO_4^- + ClO_4^-	0.16 x 10^6
ReO_4^- + Mix	0.22 x 10^6

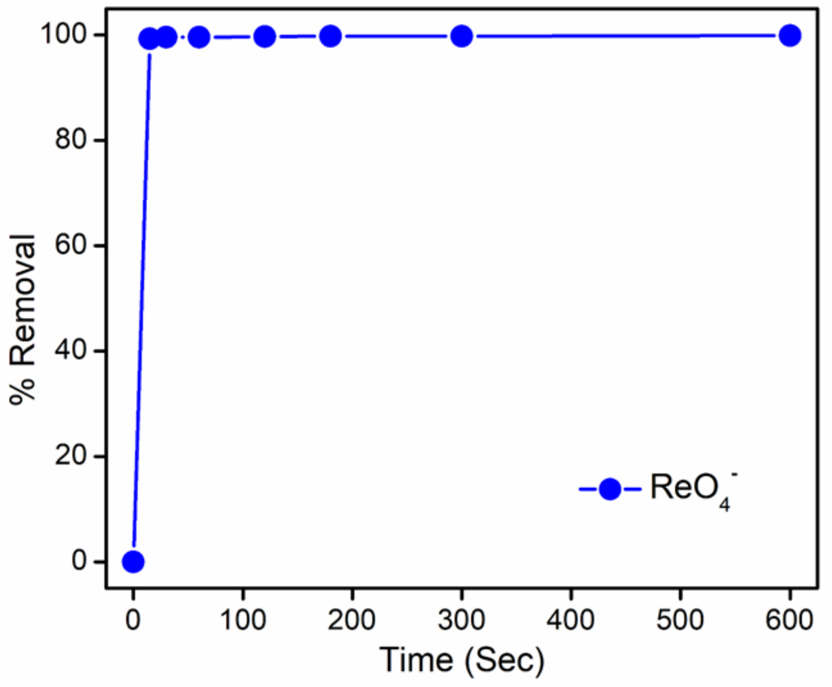

Fig. S33: Low concentration (~1000 ppb) ReO_4^{-} sorption kinetic of IPcomp-8.

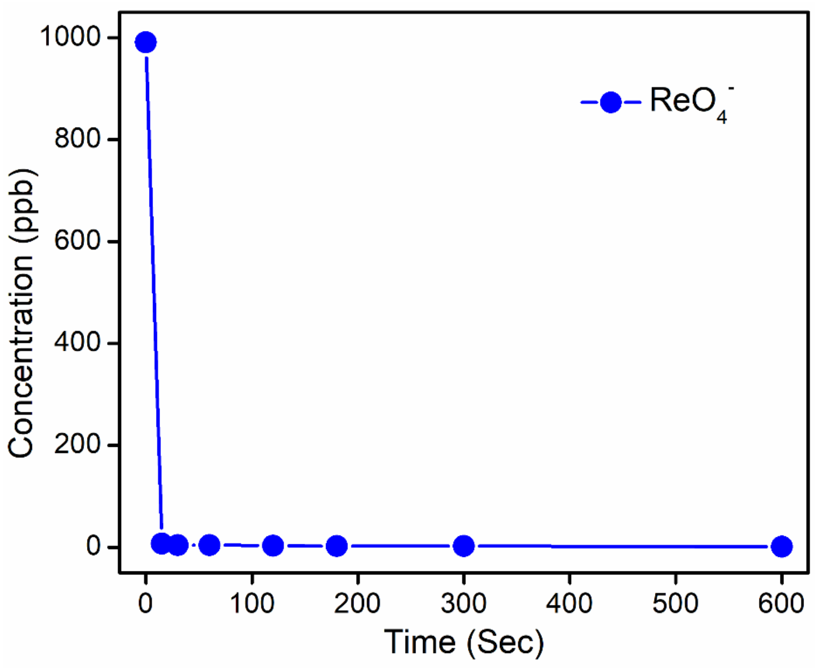

Fig. S34: Concentration decrease vs time for low concentration (~1000 ppb) ReO_4^{-} sorption kinetic of IPcomp-8.

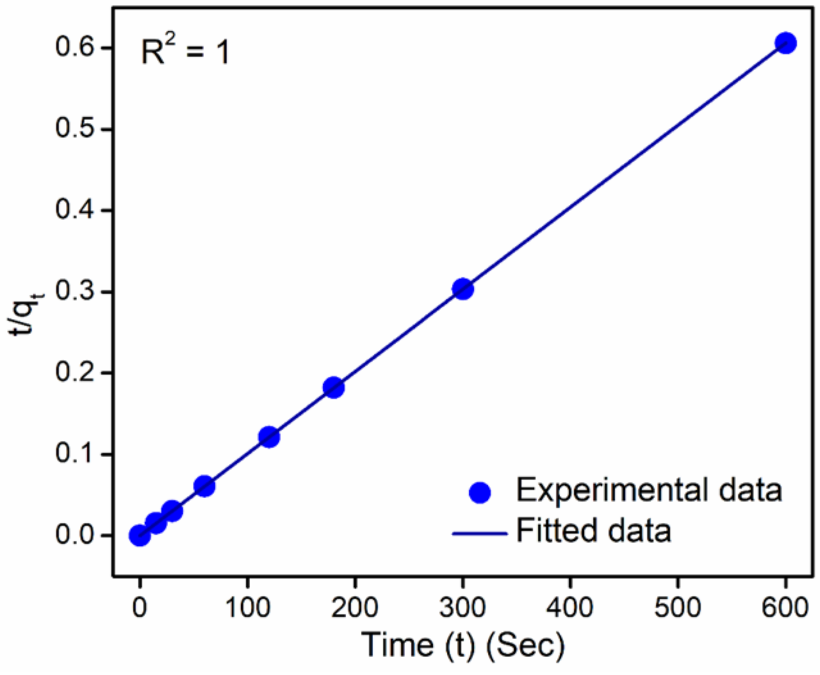

Fig. S35: Pseudo-second order linear model fitting curve of ReO_4^- sorption by IPcomp-8 at low concentration (~1000 ppb).

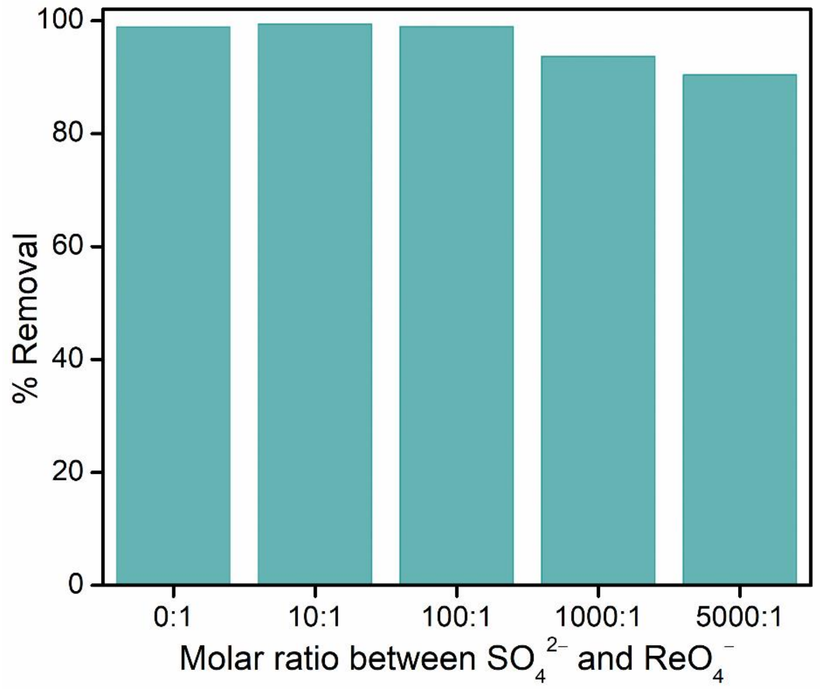

Fig. S36: Removal efficiency of IPcomp-8 towards ReO_4^- in the presence of excess SO_4^{2-} anions at low concentration.

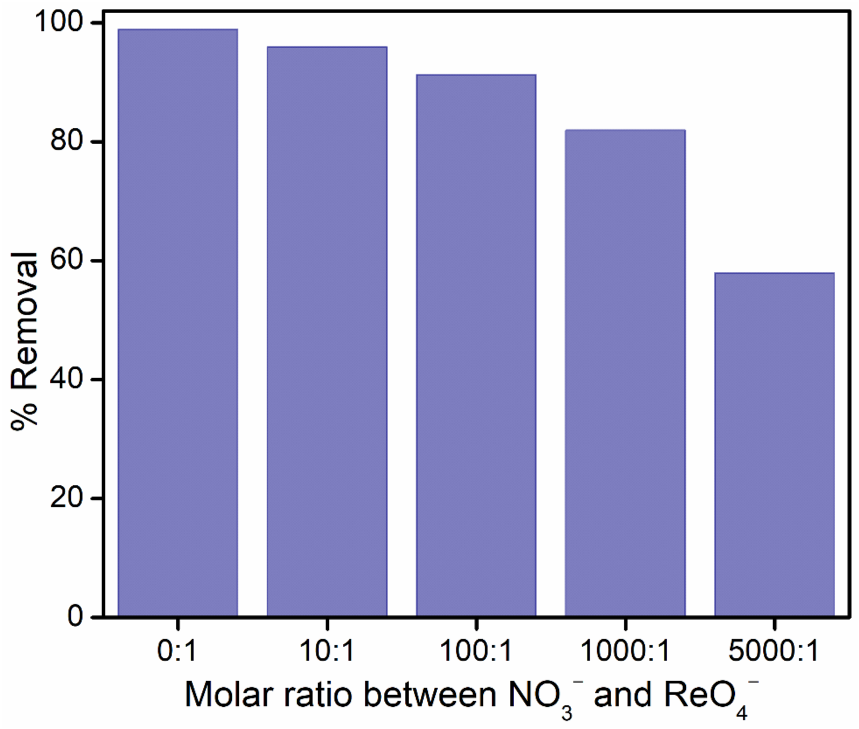

Fig. S37: Removal efficiency of IPcomp-8 towards ReO_4^- in the presence of excess NO_3^- anions at low concentration.

Section S7: Adsorption Mechanism Studies

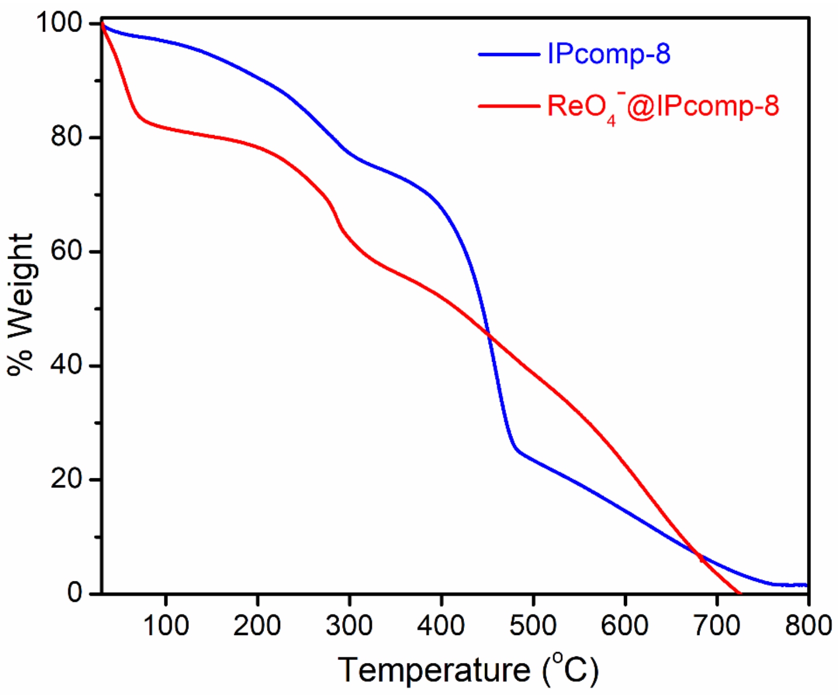

Fig. S38: TGA profile of IPcomp-8 before and after ReO_4^- treated.

Extended X-ray Absorption Fine Structure (EXAFS): The Re L3-edge (10535 eV) EXAFS measurements were performed on the scanning EXAFS beamline (BL-09) of Indus-2 SRS, RRCAT, Indore. The beamline equipped with Si (111) based double crystal monochromator for energy selection and meridional cylindrical mirror (Rh/Pt coated) for collimation. The measurements were carried out at room temperature and in transmission mode. An ion chambers were filled with N_2, He and Ar for the sample. The EXAFS measurements were carried out in the quick-XAS mode which is a continuous scan over the energy or angle range, instead of a conventional step-by-step scan during acquisition of the XAS data.[4] The second crystal of the monochromator was 60% detuned during the data collection to suppress the higher harmonic components. The standard normalization and background subtraction procedures were executed using the Athena and Artemis software version 0.9.26 to obtain normalized spectra.[5] Fourier transformed (FT) of EXAFS oscillations were calculated to observe the $|\chi(R)|$ vs R space spectra and its fitting was done using FEFF6 and ATOMS[6] programs to simulate the theoretical scattering paths according to crystallographic structure.

Table S8: EXAFS fitting parameters at the Re L3-edge for sample.

Sample	Shell	N	R (Å)	σ^2 (Å^2)	ΔE_0 (eV)	R factor
CHNOZrClRe	Re-O	4	1.734 +/- 0.023	0.0002 +/- 0.0012	6.360 +/- 3.600	0.0195

N: coordination number; R: bond distance; σ2: Debye-Waller factor; ΔE0: the inner potential correction. R factor: goodness of fit. S02 was set as 0.79.

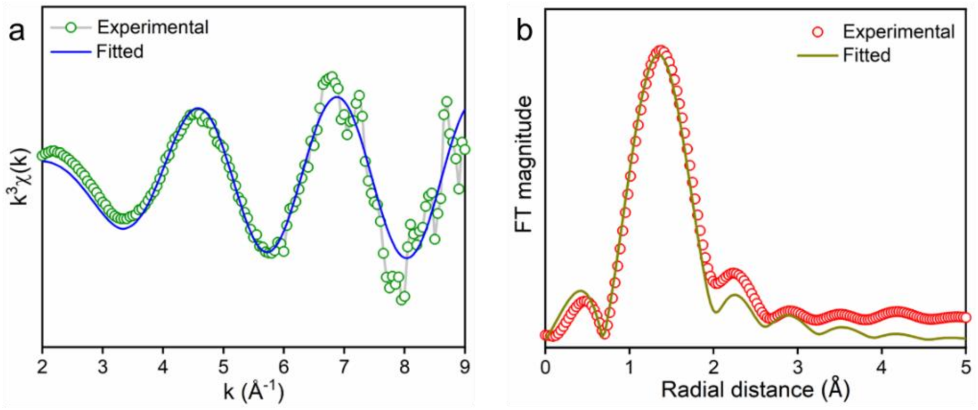

Fig. S39: (a) Raw and (b) Fourier transforms of Re L3 edge weighted EXAFS spectra of ReO_4^- treated IPcomp-8.

Supporting note 3: X-ray absorption spectroscopy (XAS) was further applied in order to investigate the stable existence of the Zr-MOP within the iCOF matrix of IPcomp-8. The local structural details around the Zr metal atoms and to calculate the oxidation state of Zr atom in the IPcomp-8 after oxoanion capture studies were investigated by X-ray absorption near edge structure (XANES) and extended X-ray absorption fine structure (EXAFS) analysis. The Zr K-edge (17998 eV) XAS spectra of ReO_4^- treated IPcomp-8 sample was compared with the standards, Zr-foil and ZrO_2 as shown in Fig. S37. The normalized XANES spectra shows a comparison of the standardes Zr-foil, ZrO_2 and the sample (Fig. S40). The appearance of the normalized XANES spectra of the sample was found to very similar to the spectra of ZrO_2. This observation confirms that the phase of Zr metal atoms in the sample is similar with that of ZrO_2 standard. The main K-edge position of Zr-sample was found to similar with ZrO_2, whereas, at a higher energy than the Zr-foil standard. This observation further indicated that the Zr atoms in sample are in Zr^{4+} oxidation state. Furthermore, k^3-weighted EXAFS spectra and its corresponding Fourier transforms (FT) spectra of Zr-sample compared with Zr-foil and ZrO_2 standards represents a pseudo radial distribution function of the Zr metal atoms (Fig. S41, S42). The various contribution from Zr-Zr, Zr-O and Zr-C correlations in sample and standards are presented in the Table S9.[7] These correlation values for the samples were found to vary close with ZrO_2 standard than that of Zr-foil. This analysis confirms the local structural environment of Zr atoms of the sample is similar to that of ZrO_2. All these XANES and EXAFS results indicated the Zr^{4+} state of the metal in the composite (IPcomp-8) after ReO_4^- capture

test, which further validate the stable existence of the MOP within the iCOF matrix of the composite.

Table S9: EXAFS fitting parameters at the Zr K-edge for sample with standards.

N: coordination number; *R*: bond distance; σ^2: Debye-Waller factor; ΔE_0: the inner potential correction. *R* factor: goodness of fit.

Sample	Shell	*N*	*R* (Å)	σ^2 (Å^2)	ΔE_0 (eV)	*R* factor
Zr foil	Zr-Zr1	12	3.218 +/- 0.029	0.0081 +/- 0.0006	-4.598 +/- 0.545	0.0199
	Zr-Zr2	6	4.484 +/- 0.025	0.0151 +/- 0.0042		
ZrO_2	Zr-O1	2	2.083 +/- 0.062	0.0007 +/- 0.0021	-3.392 +/- 1.713	0.0198
	Zr-O2	2	2.246 +/- 0.021	0.0002 +/- 0.0021		
	Zr-Zr1	4	3.581 +/- 0.176	0.0134 +/- 0.0060		
	Zr-Zr2	2	3.460 +/- 0.040	0.0040 +/- 0.0018		
CHNOZrClRe	Zr-O	2	2.263 +/- 0.093	0.0123 +/- 0.0141	-5.184 +/- 1.234	0.0085
	Zr-C1	2	2.768 +/- 0.412	0.0032 +/- 0.0029		
	Zr-Zr	2	3.481 +/- 0.133	0.0061 +/- 0.0022		
	Zr-C2	2	2.240 +/- 0.272	0.0014 +/- 0.0031		
	Zr-C3	2	2.143 +/- 0.412	0.0032 +/- 0.0029		

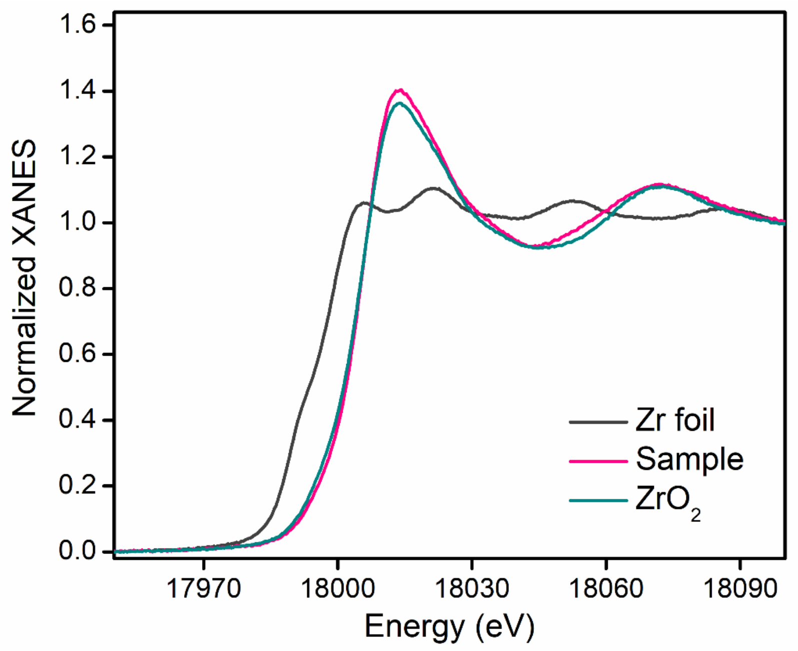

Fig. S40: EXAFS spectra of Zr K edge for ReO_4^- treated IPcomp-8.

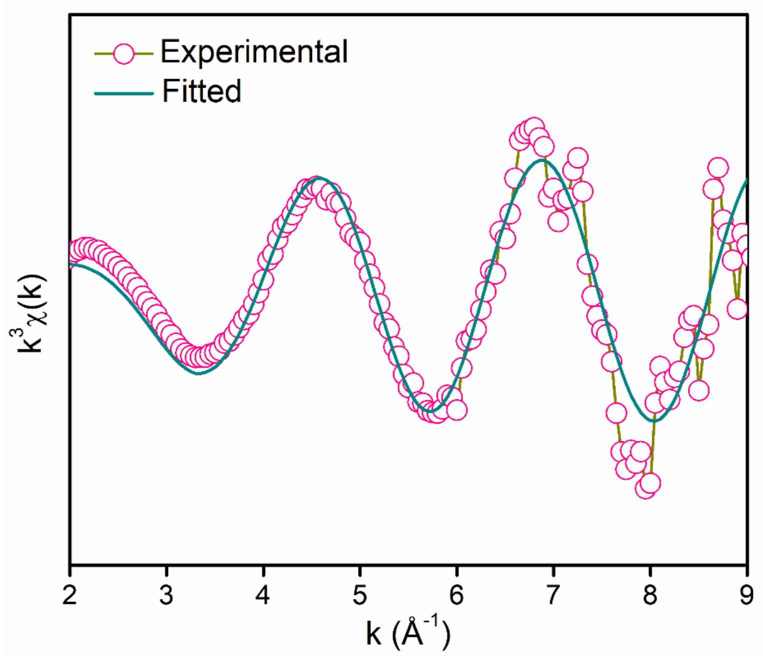

Fig. S41: Raw Zr K-edge weighted EXAFS spectra of ReO_4^- treated IPcomp-8.

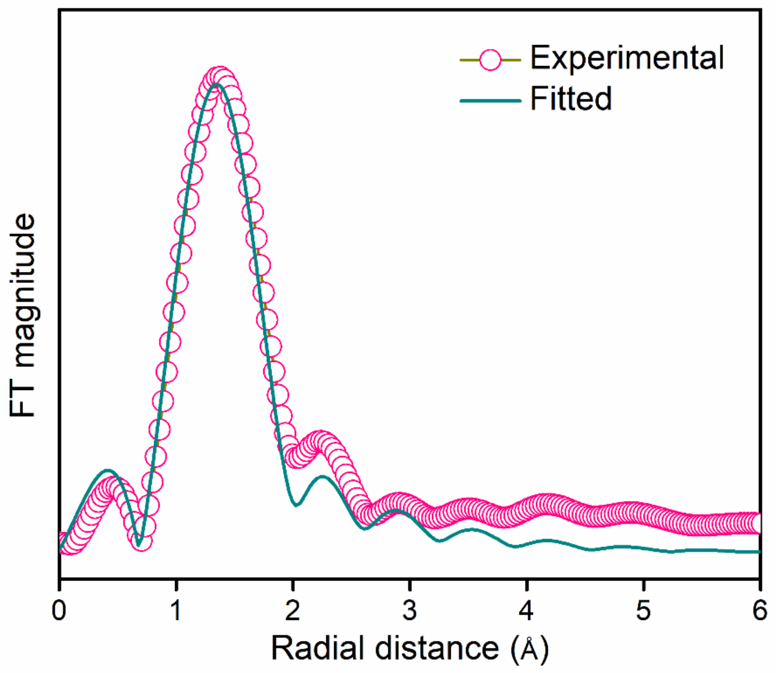

Fig. S42: Fourier transforms of Zr K edge weighted EXAFS spectra of ReO_4^- treated IPcomp-8.

Supporting note 4: Isothermal Titration Calorimetry (ITC) Experiment: Further, beyond the XPS, EXAFS and other experimental investigations, we have also performed isothermal titration calorimetry (ITC) for the thermodynamic validation of such favourable interactions between the targeted oxoanion (ReO_4^-) and the hybrid composite material (IPcomp-8) in aqueous medium. ITC experiment quantitatively provides the change in enthalpy, entropy and Gibbs free energy associated with the adsorption followed by the ion-exchange process between adsorbent and adsorbate. In this typical experiment, first, the reference cell was filled with deionized water, and a homogeneous aqueous suspension of 1 mmol adsorbate (IPcomp-8) (which was grounded into fine powder) was poured into the sample cell and the 100 ppm concentration of adsorbent (ReO_4^-) aqueous solution was placed into the syringe, while the complete mixture was stirring at 750 rpm. The first injection of each titration was fixed at 0.4 μL. The analyte concentration was determined by ICP-MS. At least two titrations for each analyte-compound pair were collected. Data analysis was performed in y. MicroCal PEAQ-ITC analysis software. The heats of injection for blank titrations [analyte ReO_4^- into blank] were subtracted from the heats of injection into compound suspensions. The recorded thermogram demonstrated a high association constant with the exothermic binding event along with the thermodynamic parameters. This result indicated the adsorption of ReO_4^- by IPcomp-8 was thermodynamically favourable as the Gibbs free energy of the process was found to be negative with positive association constant (K_a) and negative entropy. Thus, these ITC data clearly indicated the similarities between the thermodynamic parameters and the bulk-scale experimental data of strong interactions of ReO_4^- with IPcomp-8 in water.

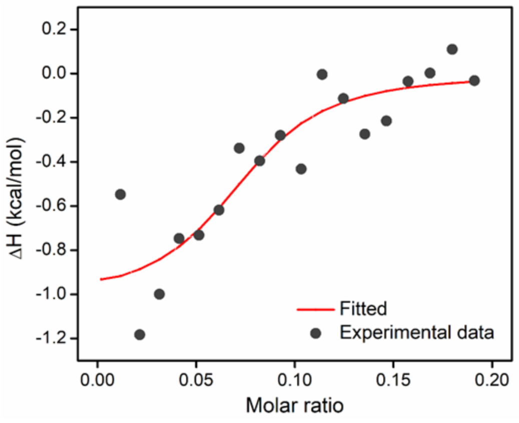

Fig. S43: Heat of injection data of ReO_4^- sorption by IPcomp-8.

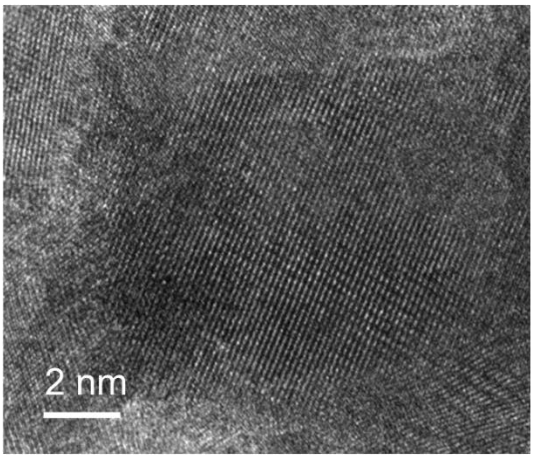

Fig. S44: HRTEM image of ReO_4^- treated IPcomp-8.

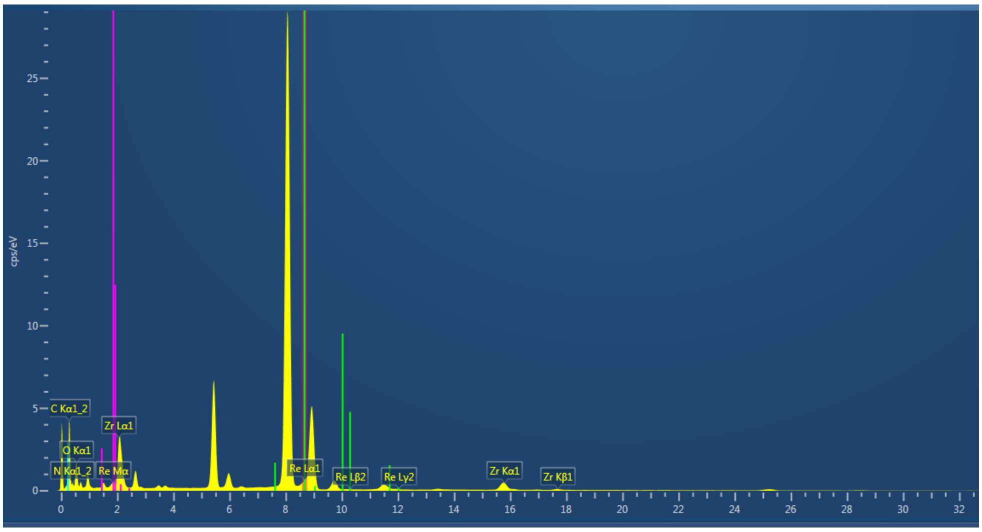

Fig. S45: EDX data of ReO_4^- treated IPcomp-8.

Section S8: Theoretical calculations studies.

The molecular-level interactions between the amino functionalized cationic MOP and oxoanion, ReO_4^-/TcO_4^-, were simulated using Discovery Studio 2017 (BIOVIA, Dassault Systèmes).[8] The DFT simulation was performed at fine quality calculation level. At first, using DMOL3 and B3LYP hybrid function the single unit of the MOP was fully geometry relaxed with following parameters: Multiplicity factor to Auto mode, Double Numeric Plus Polarizing (DNP+) basis set with water as surrounding solvent. Using simulated annealing technique structural simulation on the geometry relaxed MOP structure was realized to find a feasible interaction site of the ReO_4^-/TcO_4^- within the relaxed MOP unit. The electrostatic potential (ESP) on the van der Waals (VDW) surfaces (isodensity = 0.001 a.u.) of MOP unit was derived from ground-state electron density of the system. The Static binding energies (ΔE) at 0 K in vacuum were calculated using the following expression:

$$\Delta \mathbf{E} = \mathbf{E}_{\mathbf{MOP+Anion}} - \mathbf{E}_{\mathbf{MOP+}} - \mathbf{E}_{\mathbf{Anion}}$$

where $\mathbf{E}_\mathbf{x}$ refers, respectively, to the total energies of the MOP + Anion complex, the charged MOP+ alone, and the anion molecule respectively.

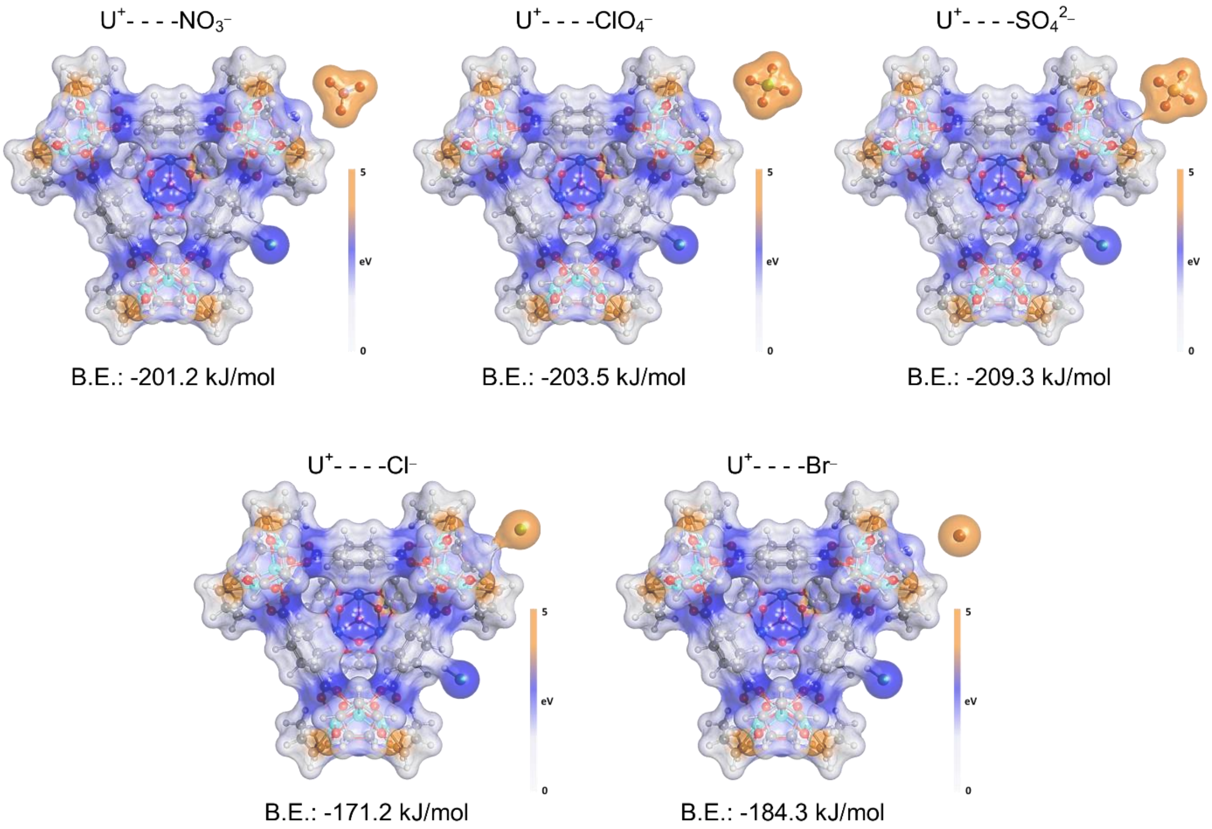

Fig. S46: Energy surface potential (ESP) diagram with their corresponding binding energies of interaction between Zr(IV)-SBU of the MOP and anions such as NO_3^-, ClO_4^-, SO_4^{2-}, Cl^-, Br^-.

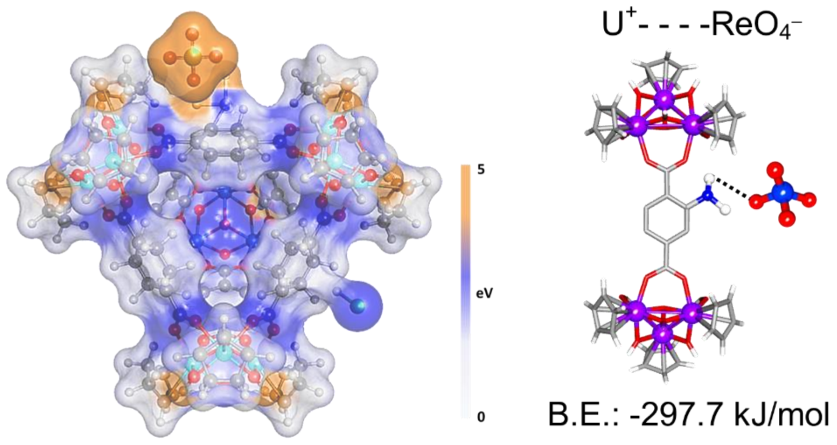

Fig. S47: Energy surface potential (ESP) diagram with binding energies of interaction between $-NH_2$ group of the MOP and ReO_4^- anion.

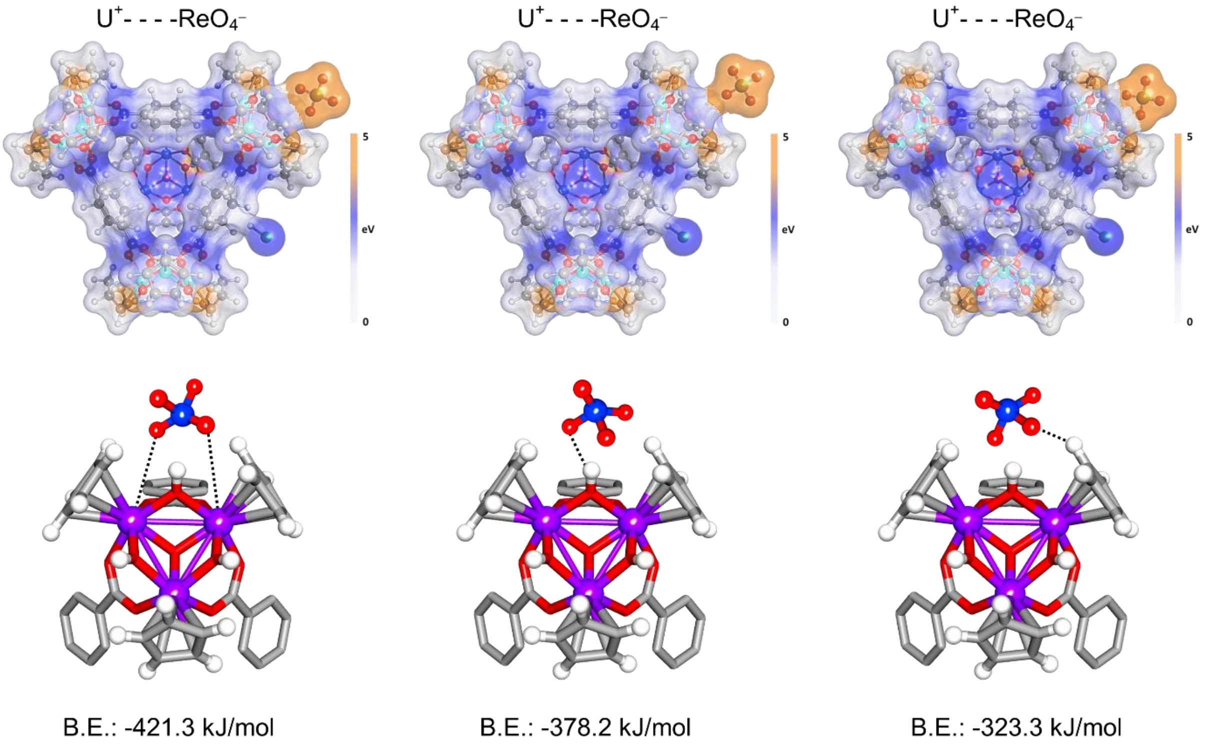

Fig. S48: Energy surface potential (ESP) diagram with binding energies of interaction between hydroxy group and hydrogen of Cp-ring of of Zr-SBU of the MOP and ReO_4^- anion.

Section S9: Mixed Matrix Membrane based studies.

S9.1. Fabrication of IPcomp-8 based MMM:

The IPcomp-8 based mixed matrix membrane (MMM) was fabricated following a previously reported protocol.[9] Briefly, at first, the 20% w/w polysulfone (PS) dope solutions were made at room temperature with DMF as the solvent, and they were swirled for 12 hours under a dry atmosphere. A portion of IPcomp-8 was then added, and the mixture was well-stirred for a further 12 hours. IPcomp-8@PS membrane was prepared by casting method (by using knife movement was set to 10 cm.sec a gap of 250 μm). The total thickness (along with support) of the supported membrane was ~100 μm. After that the composite material IPcomp-8@PS was washed with distilled water and dried in an oven for 3 hours. After cooled down to room temperature the IPcomp-8@PS was further used for applications. The IPcomp-8 based MMM was cut into circular coupons of 1.5 cm diameter and used for the metal oxoanions capture study. The permeance analysis of IPcomp-8@PS composite membrane was carried out with a stainless-steel syringe filter holder containing circular IPcomp-8@PS coupons with a 1.52 cm^2 active area fitted on a dead-end mode cell at room temperature and an upstream pressure of 1 bar (Fig. S52). The water permeance was calculated to be ~ 63±9 $L.m^{-2}.h^{-1}.bar^{-1}$.

S9.2. Dynamic flow-through-based ReO_4^- capture study: To check the continuous ReO_4^- oxoanion removal efficiency by IPcomp-8 based MMM, an ion-exchange-based flow-through experiment was conducted by packing the hybrid membrane inside a stainless-steel syringe filter holder setup. After that, stock ~1 ppm of ReO_4^- aqueous solution along with ~100-fold excess concentration of other interfering anions (such as NO_3^-, $Cl^−$, $Br^−$, $ClO_4^−$, $SO_4^{2−}$) were mixed. Different volume of this mixture was then passed through the MMM at 1 bar pressure at room temperature. Every 5 mL of permeate was collected for the % of oxoanion extraction efficiency analysis. The concentration of ReO_4^- in these permeate solutions were then analyzed through ICP-MS analysis. The recyclability test of the MMM toward ReO_4^- capture study was performed by washing the MMM with pure water, followed by saturated Na_2SO_4 solution after each cycle. The filtrate of each cycle was collected after the capture studies and the concentration was analysed through ICP-MS analysis.

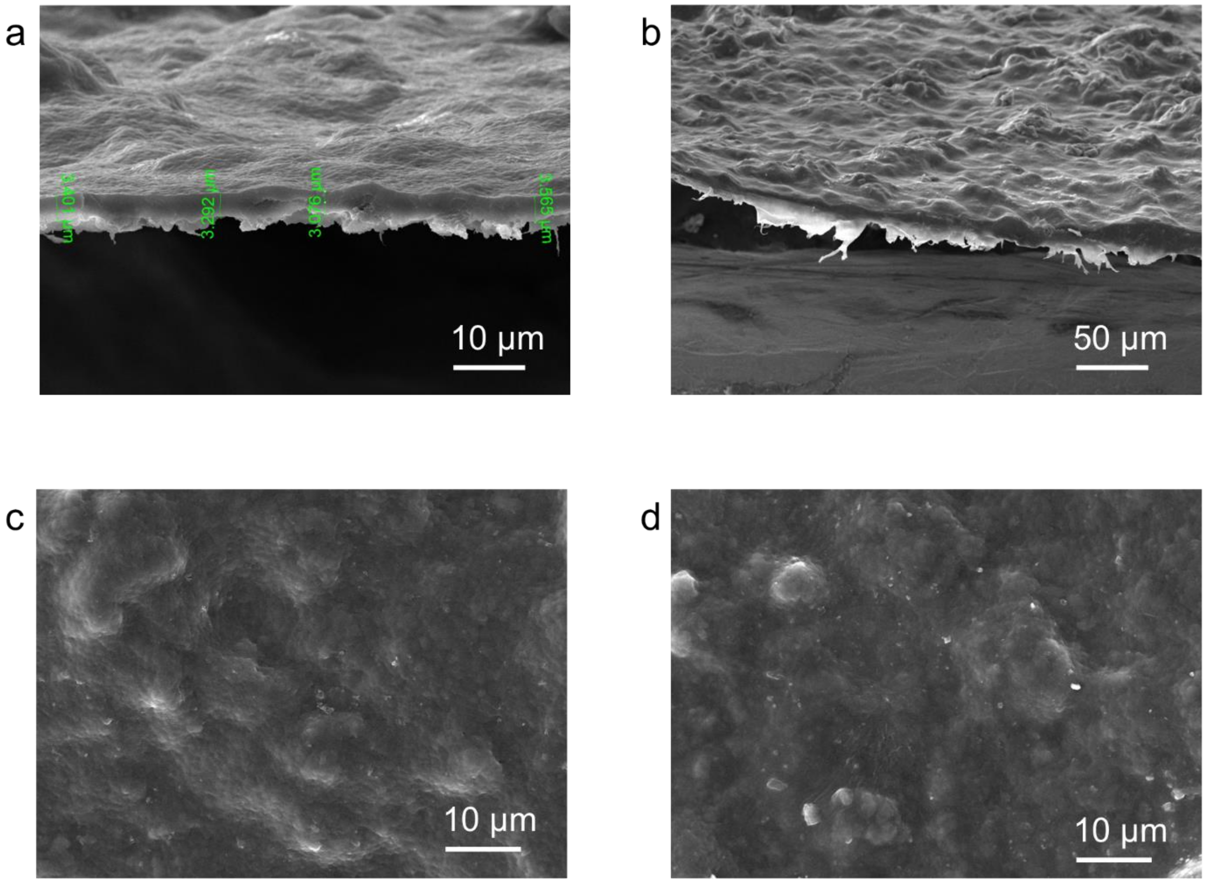

Fig. S49: FESEM (a, b) Cross-sectional and (c, d) surface morphology of IPcomp-8 MMM.

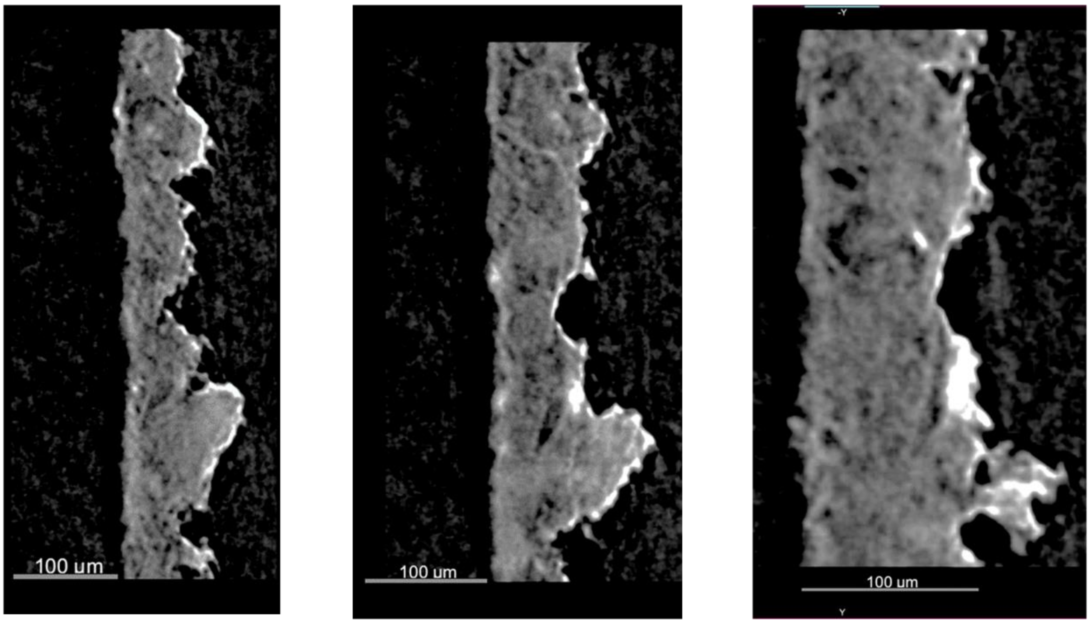

Fig. S50: Microscale X-ray computed tomography (CT) 2D cross-sectional images of IPcomp-8 MMM.

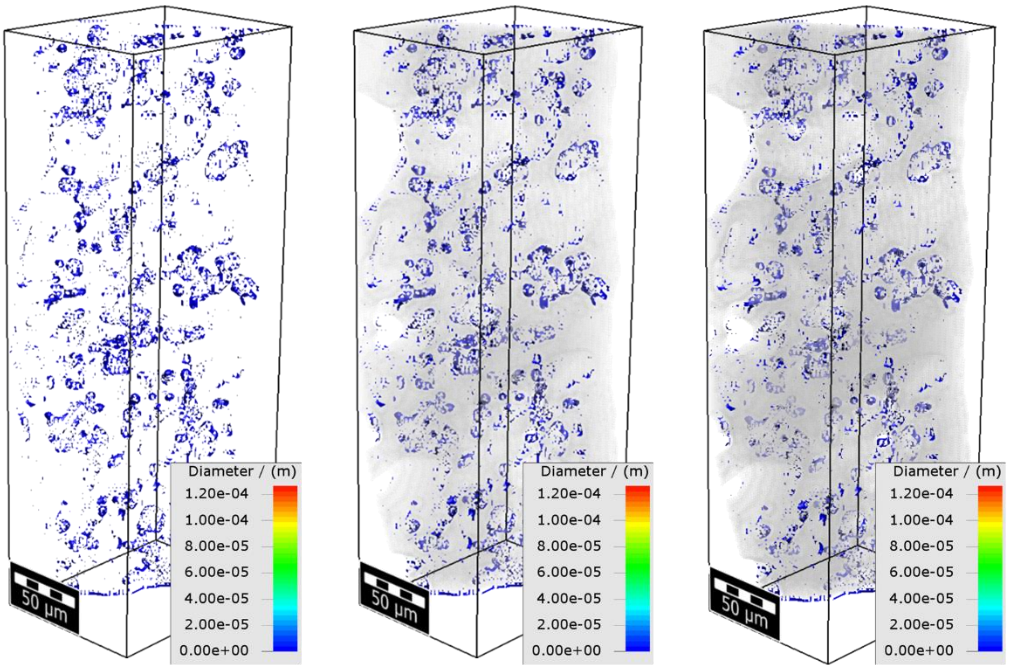

Fig. S51: Visualization of pores distribution with colour-scale CT image of IPcomp-8 MMM.

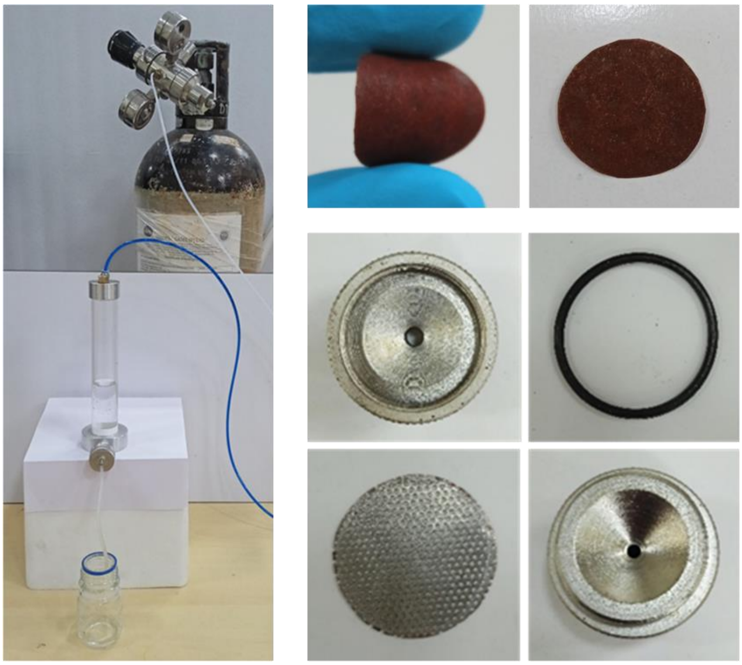

Fig. S52: Digital image of filtration assembly for metal oxoanion capture with IPcomp-8 MMM.

Section S10: References

1. G. Liu, Y. D. Yuan, J. Wang, Y. Cheng, S. B. Peh, Y. Wang, Y. Qian, J. Dong, D. Yuan and D. Zhao, *J. Am. Chem. Soc*. 2018, **140**, 6231-6234.

2. Z. Zhang, X. Dong, J. Yin, Z.-G. Li, X. Li, D. Zhang, T. Pan, Q. Lei, X. Liu, Y. Xie, F. Shui, J. Li, Yi. Mao, J. Yuan, Z. You, L. Zhang, J. Chang, H. Zhang, W. Li, Li. Fang, Li. Baiyan, X.-H. Bu and Y. Han, *J. Am. Chem. Soc.* 2022, **144**, 15, 6821–6829.

3. J. Liu, W. Duan, J. Song, X. Guo, Z. Wang, X. Shi, J. Liang, J. Wang, P. Cheng, Y. Chen, M. J. Zaworotko and Z. Zhang, *J. Am. Chem. Soc.* 2019, **141**, 12064−12070.

4. A. K. Poswal, A. Agrawal, H. K. Poswal, D. Bhattacharyya, S. N. Jha and N. K. Sahoo, *J. Synchrotron Rad.* 2016, **23**, 1-8.

5. B. Ravel and M. Newville, *J. Synchrotron Rad*. 2005, **12**, 537-541.

6. B. Ravel, J. *Synchrotron Rad*. 2001, **8**, 314-316.

7. M. Eshed, S. Pol, A. Gedanken and M. Balasubramanian, *Beilstein J. Nanotechnol*. 2011, **2**, 198–203.

8. BIOVIA, Dassault Systèmes, [Discovery Studio], [V16.1.0.15350], San Diego: Dassault Systèmes, (2017).

9. S. Mitra, S. Kandambeth, B. P. Biswal, M. A. Khayum, C. K. Choudhury, M. Mehta, G. Kaur, S. Banerjee, A. Prabhune, S. Verma, S. Roy, U. K. Kharul and R. Banerjee, *J. Am. Chem. Soc*. 2016, **138**, 2823−2828.
